# Phase‐Space Generalized Brillouin Zone For Spatially Inhomogeneous Non‐Hermitian Systems

**DOI:** 10.1002/advs.202508047

**Published:** 2025-08-11

**Authors:** Qingya Li, Hui Jiang, Ching Hua Lee

**Affiliations:** ^1^ School of Physics Dalian University of Technology Dalian 116024 China; ^2^ Department of Physics National University of Singapore Singapore 117551 Singapore

**Keywords:** generalized impurity Generalized Brillouin Zone (GBZ) treatment, non‐Hermitian skin effect in phase space, novel Generalized Brillouin Zone bifurcations, spatially inhomogeneous systems, unconventional Generalized Brillouin Zone in phase space, unconventional topological transitions

## Abstract

The generalized Brillouin zone (GBZ) is highly successful in characterizing the topology and band structure of non‐Hermitian systems. However, its applicability ischallenged in spatially inhomogeneous settings, where the non‐locality of non‐Hermitian pumping competes with Wannier‐Stark localization and quantum interference, potentially leading to highly non‐exponential state accumulation. To transcend this major conceptual bottleneck, a general phase‐space GBZ formalism is developed that encodes non‐Bloch deformations in both position and momentum space, such as to accurately represent spatially inhomogeneous non‐Hermitian pumping. A key new phenomenon is the bifurcation of the phase‐space GBZ branches, which allows certain eigenstates to jump abruptly between different GBZ solutions at various points in real space, such as to accommodate pockets of inhomogeneity. The freedom in the jump locations opens up an emergent degree of freedom that protects the stability of real spectra and, more impressively, the robustness of a new class of topological zero modes unique to GBZ bifurcation. The response from these novel spectral and GBZ singularities can be readily demonstrated in mature metamaterial platforms such as photonic crystals or circuit arrays. The framework directly generalizes to more complicated unit cells and further hoppings, opening up a vast new arena for exploring unconventional spectral and topological transitions, as well as GBZ fragmentation in spatially inhomogeneous non‐Hermitian settings.

## Graphical abstract

A novel phase‐space generalized Brillouin zone (GBZ) framework to devised captures the interplay of spatial inhomogeneity and non‐local non‐Hermitian effects. This reveals new degrees of freedom such as jumps in the bifurcated GBZ, as well as new routes for topological protection, with potential applications in characterizing inhomogeneous or disordered metamaterials.

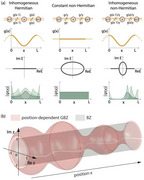

## 1 Introduction

The non‐Hermitian skin effect (NHSE) represents a very robust form of localization caused by the directed amplification from asymmetric hoppings. Such non‐Bloch behavior has led to various paradigm shifts in the way band structures are characterized, as epitomized by modified non‐Bloch topological invariants defined on the generalized Brillouin zone (GBZ).^[^ ^1^, ^2^, ^3^, ^4^, ^5^, ^6^, ^7^, ^8^, ^9^, ^10^ ^]^ Competition between more than one type of asymmetric hopping can furthermore modify critical scaling properties^[^ ^11^, ^12^, ^13^, ^14^, ^15^, ^16^, ^17^, ^18^ ^]^ and induce non‐Hermitian pseudo‐gaps^[^ ^19^ ^]^ that break the one‐to‐one correspondence between the open and periodic boundary conditions (OBC and PBC) spectra, challenging existing notions of non‐Bloch topological bulk‐boundary correspondences.^[^ ^20^, ^21^, ^22^, ^23^, ^24^, ^25^ ^]^

While most existing investigations have focused on NHSE localization against hard open boundaries,^[^ ^26^, ^27^, ^28^, ^29^, ^30^, ^31^, ^32^, ^33^, ^34^, ^35^, ^36^, ^37^, ^38^, ^39^, ^40^ ^]^ i.e., physical edges, the underlying directed pumping mechanism gives rise to localized state accumulations as long as translation symmetry is broken. Recently, it has been recognized that spatial inhomogeneities that are not abrupt cut‐offs can support other less‐known but interesting phenomena such as scale‐free eigenstates^[^ ^41^, ^42^, ^43^, ^44^, ^45^, ^46^ ^]^ and non‐Hermitian Anderson localization.^[^ ^47^, ^48^, ^49^, ^50^, ^51^, ^52^, ^53^, ^54^ ^]^ These enigmatic phenomena arise because of the nontrivial interplay between the emergent nonlocality from directed non‐Hermitian pumping, and the momentum non‐conserving spatial inhomogeneities associated with Stark localization, quantum confinement, and band flattening.^[^ ^55^, ^56^, ^57^, ^58^ ^]^ However, a unified framework encompassing the breadth of these exciting interplays does not yet exist.

To provide this unified framework, we propose in this work the new concept of phase‐space GBZs, where the complex momentum deformation for describing the state accumulation profile depends on the spatial position *x* in addition to the Bloch momentum *k*. By considering the subtle but important skin contributions from spatial hopping gradients, we obtained an analytic ansatz that accurately predicts the energy spectrum and most skin eigenstate profiles in various 1D spatial inhomogeneity profiles, from soft boundaries to impurities to physical edges. This phase‐space GBZ construction is also generalized to two‐component systems, where it is shown to accurately predict a new topological phase transition arising from the tuning of the soft boundary width.

Our phase‐space GBZ gives a firm theoretical basis for a number of new phenomena. Most salient is the bifurcation of the GBZ into various branches due to spatial inhomogeneity. OBC‐like eigenstates can exhibit branch switching in β(*x*), giving rise to spatial profiles built from multiple local decay rates. Such “inhomogeneous skin” regions also give rise to unconventional but universal spectral branching in the complex plane, beyond what is allowed by spatially homogeneous GBZs. Additionally, in multi‐component scenarios, varying the spatial hopping inhomogeneity can also drive topological phase transitions unique to NHSE‐pumped states.

## 2 Results

### 2.1 1D Monoatomic Chain with Spatially Inhomogeneous Asymmetric Hoppings

To move beyond the conventional GBZ formalism built upon translationally invariant lattices with constant but asymmetric hopping amplitudes under open boundaries,^[^ ^1^, ^2^, ^59^, ^60^, ^61^, ^62^, ^63^ ^]^ we consider systems where the hopping strengths vary spatially via a profile *g*(*x*). This modulation breaks translation symmetry even under PBCs, thus introducing nontrivial spatial effects beyond standard NHSE descriptions.

(1) $$\begin{array}{cc} H & =\sum_{x=1}^{L-1}g\left(x\right)\left(\frac{1}{\gamma }\left|x\right\rangle \left\langle x+1\right|+\gamma \left|x+1\right\rangle \left\langle x\right|\right)\\ & \;\;+g\left(L\right)\left(\frac{1}{\gamma }\left|L\right\rangle \left\langle 1\right|+\gamma \left|1\right\rangle \left\langle L\right|\right) \end{array}$$

on a ring with *L* sites, where *g*(*x*)/γ and *g*(*x*)γ are the left and right hoppings between sites *x* and *x* + 1, respectively. Here, we have fixed the local hopping asymmetry γ to a constant value, since any desired spatial profile of the hopping asymmetry γ(*x*) can be easily obtained from Equation (1) via a local basis transformation $\left|x\right\rangle \to \gamma^{-x}\Pi_{x^{\prime }=1}^{x-1}\gamma \left(x^{\prime }\right)\left|x\right\rangle$, $\left\langle x\right|\to \left\langle x\right|\gamma^{x}/\Pi_{x^{\prime }=1}^{x-1}\gamma \left(x^{\prime }\right)$, with γ set to ${\left(\Pi_{x^{\prime }=1}^{L}\gamma \left(x^{\prime }\right)\right)}^{1/L}$. PBCs are used instead of OBCs, such that the only source of spatial inhomogeneity is from *g*(*x*).

**Figure 1:**
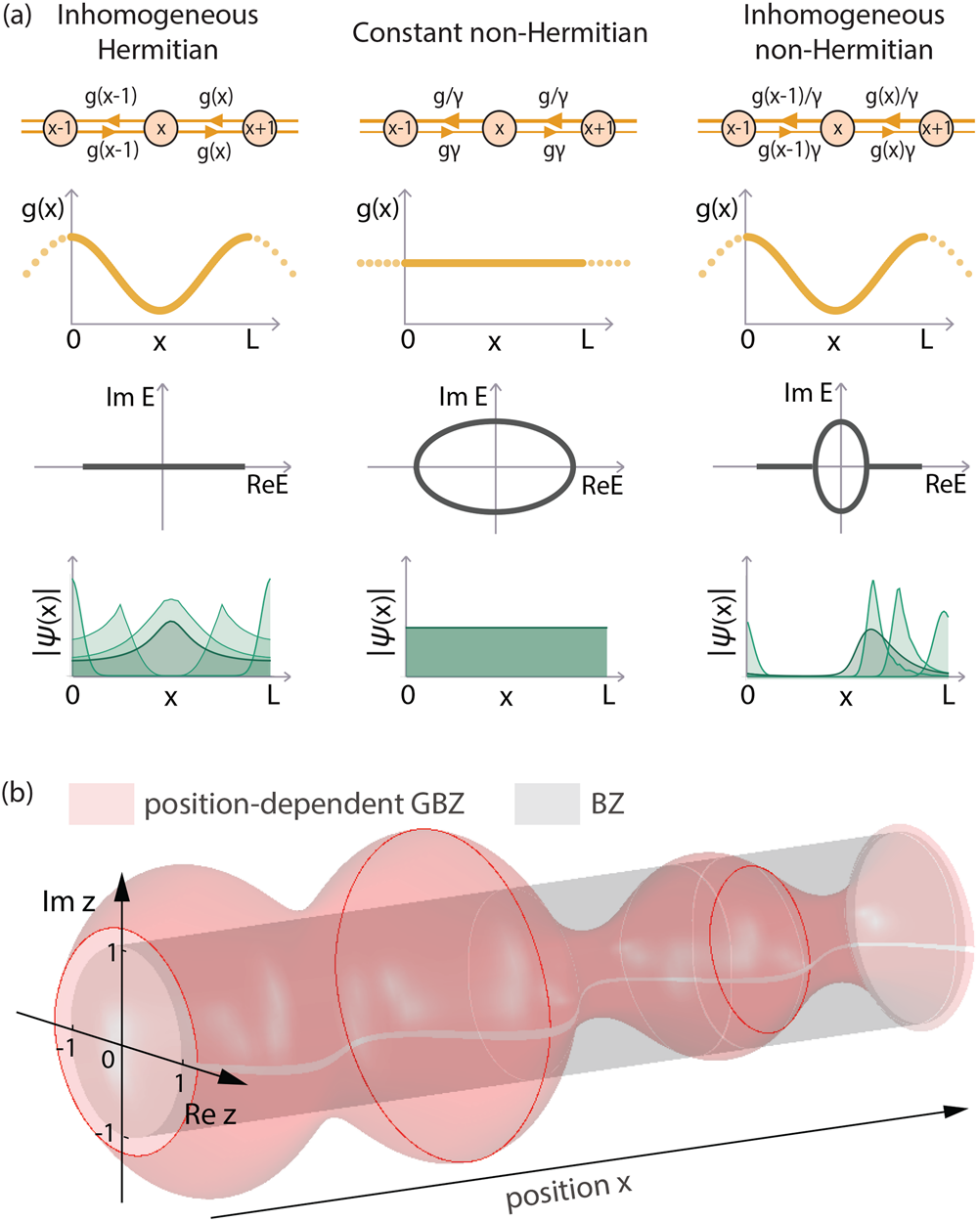
Non‐trivial Interplay of spatial inhomogeneity and anisotropy in the lattice hoppings. a) Left: Spatial inhomogeneity in the lattice hoppings (with profile given by *g*(*x*)) leads to inhomogeneities in the eigenstates ψ(*x*) that are locally proportional to $1/\sqrt{g(x)}$. Center: Without spatial hopping inhomogeneity, i.e., PBCs with constant *g*(*x*), the eigenstates exhibit constant magnitudes despite non‐Hermitian hopping anisotropy γ (asymmetric hoppings). Right: With the simultaneous presence of spatial inhomogeneity and asymmetry in the hoppings, new tails branch out from the eigenspectrum and the wavefunctions accumulate asymmetrically against *g*(*x*) troughs, which behave like “partial” boundaries. b) An inhomogeneous non‐Hermitian hopping strength profile *g*(*x*) results in a position‐dependent generalized Brillouin zone (GBZ) *z*_*n*_(*x*) = ψ_*n*_(*x* + 1)/ψ_*n*_(*x*) defined in phase space, as given in Equation (4), such that the wavefunctions exhibit different spatial decay or growth rates at different positions. Shown is the GBZ for an illustrative *g*(*x*) = (sin (4π*x*/*L*) · cos (2π*x*/*L*) + 1.2)^−1^ with γ = 0.2.

The PBC ansatz Hamiltonian Equation (1) encompasses the usual well‐studied limits as special cases, as we first schematically describe in the following. For γ = 1 [**Figure** **1**a (Left)], it reduces to a Hermitian nearest‐neighbour tight‐binding chain with real spectrum and eigenstates ψ(*x*) that depend only locally on the texture *g*(*x*). For constant *g*(*x*) = *g* with non‐Hermitian γ ≠ 1 [Figure 1a (Center)], it reduces to the usual Hatano‐Nelson model^[^ ^64^ ^]^ with a complex elliptical spectrum. Even though the eigenstates ψ(*x*) are pumped leftwards by the hopping asymmetry, they do not have anywhere to accumulate against due to the PBCs and uniform *g*(*x*), and thus exhibit spatially uniform amplitudes |ψ(*x*)|.

But with the simultaneous presence of nontrivial hopping asymmetry γ ≠ 1 and non‐constant spatial hopping profile *g*(*x*) [Figure 1a (Right)], the non‐Hermitian pumped eigenstates are able to accumulate partially against the *g*(*x*) inhomogeneities. As schematically shown, illustrative eigenstates generally accumulate toward the right of the *g*(*x*) minimum, which is at the center. These spatial inhomogeneities invalidate the standard exponential description of NHSE eigenstates, necessitating a more general, position‐dependent GBZ formalism. Also, as compared to the spectrum in Figure 1a (Center), the inhomogeneity in *g*(*x*) has given rise to additional spectral branches at the side, reminiscent of (but distinct from) the OBC spectra of uniform models with asymmetric hoppings.^[^ ^11^, ^13^, ^14^, ^63^, ^65^, ^66^, ^67^, ^68^ ^]^

### 2.2 Phase‐Space Generalized Brillouin Zone (GBZ) for Inhomogeneous Single‐Component Chains

#### 2.2.1 General Formalism Setup

To rigorously characterize the anomalous non‐local consequences from spatially non‐uniform *g*(*x*), we examine the Schrödinger eigen‐equation $H|\psi_{n}\rangle =E_{n}|\psi_{n}\rangle$ of Equation (1):

(2) $$\begin{array}{c} \frac{g(x)}{\gamma }\psi_{n}\left(x+1\right)+g\left(x-1\right)\gamma \psi_{n}\left(x-1\right)=E_{n}\psi_{n}\left(x\right) \end{array}$$

where *E*_*n*_ is the eigenenergy of the eigenstate $|\Psi_{n}\rangle =\sum_{x}\psi_{n}\left(x\right)\left|x\right\rangle$. For later convenience, we define the lower and upper bounds of *g*(*x*) by *g*_min_ = Min[*g*(*x*)] and *g*_max_ = Max[*g*(*x*)], such that *g*_min_ = *g*_max_ only if the hoppings are completely homogeneous in space.

Since *g*(*x*) acts like an energy rescaling factor in the Hermitian continuum limit, local energy conservation requires that *g*(*x*)|ψ_*n*_(*x*)|^2^ remains invariant. Extending this to generic non‐Hermitian cases where non‐local pumping arises from γ ≠ 1, we propose an ansatz for the state amplitude ψ_*n*_(*x*) at site *x* to be

(3) $$\begin{array}{c} \psi_{n}\left(x\right)=\frac{1}{\sqrt{g(x)}}\gamma^{x}\prod_{x^{\prime }=1}^{x}\beta_{n}\left(x^{\prime }\right) \end{array}$$

where the γ^*x*^ keeps track of the pure exponential state accumulation, and $\prod_{x^{\prime }=1}^{x}\beta_{n}\left(x^{\prime }\right)$ denotes the part of the state accumulation that depends specifically on the *g*(*x*) profile, which is the key quantity that we will focus on in this work.

Together, γ and β_*n*_(*x*) define the *phase‐space* GBZ, as schematically illustrated in Figure 1b:

(4) $$\begin{array}{c} z_{n}\left(x\right)=\frac{\psi_{n}\left(x\right)}{\psi_{n}\left(x-1\right)}=\sqrt{\frac{g(x-1)}{g(x)}}\gamma \beta_{n}\left(x\right) \end{array}$$

which is defined in position‐momentum phase space, depending both on the position *x*, and the state momentum^[^ ^69^ ^]^ indexed through *n*. The quantities *z*_*n*_(*x*), β_*n*_(*x*), and the associated decay rate κ_*n*_(*x*) = −log|β_*n*_(*x*)| will be used repeatedly in later sections without redefinition. Different from the usual spatially homogeneous (i.e., constant *g*(*x*) = *g*) *OBC* case, where we simply have *z*(*x*) = γ, we have the new β_*n*_(*x*) factor that we call the phase‐space GBZ factor, which would compensate for the usual γ rescaling factor in a homogeneous PBC system. Its spatial periodicity β_*n*_(*x* + *L*) = β_*n*_(*x*) is inherited from that of the hopping amplitude profile *g*(*x*) and the state amplitude ψ_*n*_(*x*).

To determine the form of the phase‐space GBZ factor β_*n*_(*x*), we substitute this ansatz [Equation (3)] into the bulk equation Equation (2) and arrive at

(5) $$\begin{array}{c} \frac{\sqrt{g(x)}g\left(x\right)}{\sqrt{g(x+1)}}\beta_{n}\left(x+1\right)+\frac{\sqrt{g(x)g(x-1)}}{\beta_{n}\left(x\right)}=E_{n} \end{array}$$

This expression Equation (5) applies to generic hopping inhomogeneity *g*(*x*), even discontinuous ones to a good approximation (see Section Handling Isolated Hopping Discontinuities). However, for most cases that we shall consider, we will further make the key assumption of local spatial continuity: If we consider a sufficiently smooth hopping function *g*(*x*) such that the spatial gradients satisfy

(6) $$\begin{array}{cc} & g^{\prime }\left(x\right)\ll g\left(x\right) \end{array}$$

(7) $$\begin{array}{cc} & \beta_{n}^{\prime }\left(x\right)\ll \beta_{n}\left(x\right) \end{array}$$

in the thermodynamic limit of large *L*, Equation (5) simplifies to

(8) $$\begin{array}{ccc} & \beta_{n}\left(x+1\right)+\frac{1}{\beta_{n}\left(x\right)}=\frac{E_{n}}{g(x)} & \end{array}$$

(9)

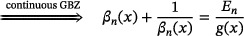

The second line, obtained by assuming local continuity of the phase‐space GBZ, decouples the inter‐dependency between neighbouring β_*n*_(*x*) and β_*n*_(*x* + 1), such that β_*n*_(*x*) can be solved solely from the local hopping *g*(*x*) and the eigenenergy *E*_*n*_. In practice, the locally continuous GBZ assumption can be justified a posteriori by comparing its analytic predictions with numerical diagonalization results. From our results presented later, it turns out that even the rapidly oscillating phases in the wavefunctions do not compromise this assumption, at least for the majority of the reasonably smooth eigenstates.

From Equation (9), the phase‐space GBZ factor β_*n*_(*x*) can be directed solved in terms of the eigenenergy *E*_*n*_ and the spatial hopping amplitude profile *g*(*x*), with a pair of solutions β_*n*, ±_(*x*) given by

(10) $$\begin{array}{cc} \beta_{n,\pm }\left(x\right)= & \exp\left(\pm \cosh^{-1}\left(\frac{E_{n}}{2g(x)}\right)\right)\\ = & \frac{E_{n}}{2g(x)}\pm i\sqrt{1-\frac{E_{n}^{2}}{4g^{2}\left(x\right)}}\; \end{array}$$

with |β_*n*, +_| ⩾ 1. To keep track of the extent of spatial state accumulation, we decompose $\beta_{n,\pm }$ as

(11) $$\begin{array}{cc} \beta_{n,\pm }\left(x\right) & =e^{ik_{n,\pm }\left(x\right)}e^{-\kappa_{n,\pm }\left(x\right)} \end{array}$$

where

(12) $$\begin{array}{c} \kappa_{n,\pm }\left(x\right)=-\log\left|\beta_{n,\pm }\left(x\right)\right|=\mp \text{Re}\left[\cosh^{-1}\left(\frac{E_{n}}{2g(x)}\right)\right]\; \end{array}$$

represents the local contribution to the inverse inhomogeneous skin depth at position *x*, and the phase *k*_*n*, ±_(*x*) describes the effective Bloch‐like phase oscillations with spatially varying wavenumber *dk*_*n*, ±_(*x*)/*dx*.

#### 2.2.2 Spatially Inhomogeneous Generalized Brillouin Zone (GBZ) Branches

Although Equation (10) or Equation (12) may look superficially similar to that of the usual Hatano‐Nelson model,^[^ ^64^ ^]^ where *g*(*x*) is constant, the inhomogeneity of *g*(*x*) brings about various new levels of subtleties. First, labeling the β_*n*, ±_(*x*) solutions such that

(13) $$\begin{array}{c} |\beta_{n,+}\left(x\right)|\geq 1\geq |\beta_{n,-}\left(x\right)| \end{array}$$

We identify the following distinct regions in real space *x*:

- **Pure skin region**: |β_*n*, +_(*x*)| = |β_*n*, −_(*x*)| = 1, i.e., κ_*n*, ±_ = 0, such that the spatial state profile in these positions is purely exponential (just like usual non‐Hermitian skin modes), arising only from the γ^*x*^ term in Equation (3). It occurs in the region |Re(*E*_*n*_)| ⩽ 2*g*(*x*) and Im(*E*_*n*_) = 0.
- **Inhomogeneous skin region**: nonconstant |β_*n*, +_(*x*)| > 1 > |β_*n*, −_(*x*)|, such that the spatial state profile is manifestly non‐exponential. It occurs when Im(*E*_*n*_) ≠ 0 or |Re(*E*_*n*_)| > 2*g*(*x*), the latter which represents pockets of weak hopping with no spatially homogeneous analog.

Importantly, the same physical eigenstate ψ_*n*_(*x*) can exhibit both pure skin and inhomogeneous skin behaviors at different locations *x*. But, whether exhibiting pure or inhomogeneous skin, ψ_*n*_(*x*) can only incorporate one of the two possible β_*n*, ±_(*x*) solution branches at any particular point *x*.

We define the choice function σ(*x*) that takes values of ±1 depending on which branch is chosen at position *x*; exactly how σ(*x*) can be determined will be detailed in the next subsection. Notating the chosen branch as β_*n*_(*x*) = β_*n*, σ(*x*)_(*x*) with branch selection function σ(*x*) = ±1, the decay rate becomes position‐dependent

(14) $$\begin{array}{c} \kappa_{n}\left(x\right)=-\sigma \left(x\right)\text{Re}\left[\cosh^{-1}\left(\frac{E_{n}}{2g(x)}\right)\right] \end{array}$$

extending the definition in Equation (12). We distinguish between two different scenarios for the phase‐space GBZ:

✴ **Continuous phase‐space GBZ**: Either β_*n*_(*x*) = β_*n*, +_(*x*) or β_*n*_(*x*) = β_*n*, −_(*x*) for all *x*, such that only one branch is ever realized, i.e., σ(*x*) = ±1 for all *x*.
✴ **Discontinuous phase‐space GBZ**: β_*n*_(*x*) switches (jumps) between the β_*n*, +_(*x*) and β_*n*, −_(*x*) branches at the so‐called GBZ inversion points *x*_jump_, which exist due to the spatial inhomogeneity from *g*(*x*).

In a nutshell, the phase‐space GBZ connectivity can be classified by the number of *x*_jump_ points where σ(*x*) alternates between +1 and −1. An even number of alternations must occur since σ(*x*) is periodic in *x* and has to switch an even number of times. A continuous/discontinuous phase‐space GBZ corresponds to a zero/nonzero number of *x*_jump_ points – in this work, we shall explicitly examine only cases with at most two *x*_jump_ points, since more complicated cases can be broken down into multiple discontinuous GBZs in real space and analyzed separately.

Physically, we can understand the need to join the GBZ solution branches by regarding spatial inhomogeneities as “soft” defect regions. On either side of a spatial defect, we expect the eigenstates to fall off in opposite directions, i.e., take on opposite κ_*n*_. But since the inhomogeneous pockets span over extended regions, the exact position of the solution jump is not fixed, and constitutes a new degree of freedom in the full eigensolutions.

Note that κ_*n*_(*x*) controls the eigenstate amplitude not just locally at *x*, but, in fact, non‐locally

(15) $$|\psi_{n}\left(x\right)|=\frac{\gamma^{x}}{\sqrt{g(x)}}\prod_{x^{\prime }=1}^{x}e^{-\kappa_{n}\left(x^{\prime }\right)}$$

which, in terms of the spatial gradient of the state amplitude, takes the form

(16) $$\frac{d}{dx}\left(\log(\sqrt{g(x)}\left|\psi_{n}\left(x\right)\right|)\right)=\log\gamma -\kappa_{n}\left(x\right)$$

As such, κ_*n*_(*x*) can be interpreted as a position‐dependent correction that compensates for the exponential accumulation from γ^*x*^, thus ensuring that the wavefunction satisfies PBCs. As the simplest example, consider the spatially homogeneous Hatano‐Nelson model where *g*(*x*) = *g* and the PBC energy can be

(17) $$E_{n}=g\left(\gamma e^{ip_{n}}+\gamma^{-1}e^{-ip_{n}}\right)=2g\cosh\left(\log\gamma +ip_{n}\right)$$

where real momentum *p*_*n*_ ∈ [0, 2π). We have

(18) $$\kappa_{n}\left(x\right)=\text{Re}\left(\cosh^{-1}\left(E_{n}/2g\right)\right)=\log\gamma$$

which serves to exactly cancel off the γ^*x*^ accumulation [Equation (15)] in the case of the Hatano‐Nelson model.

#### 2.2.3 Determining the Allowed Energy Spectrum

The PBC condition ψ_*n*_(*x* + *L*) = ψ_*n*_(*x*) of the system imposes an important constraint on β_*n*_(*x*) that enables its spectrum *E*_*n*_ and eigenstates ψ_*n*_(*x*) to be uniquely solved. Substituting the PBC condition into the ansatz Equation (3), we obtain

(19) $$\begin{array}{cc} & \gamma^{-L}=\prod_{x=1}^{L}\beta_{n}\left(x\right) \end{array}$$

which, from Equation (12), is equivalent to the following handy constraint on the skin depth and eigenenergies

(20) $$\begin{array}{cc} \log(\gamma )= & \frac{1}{L}\sum_{x=1}^{L}\kappa_{n}\left(x\right)\\ = & -\frac{1}{L}\sum_{x=1}^{L}\sigma \left(x\right)\;\text{Re}\left(\cosh^{-1}\left(\frac{E_{n}}{2g(x)}\right)\right). \end{array}$$

**Figure 2:**
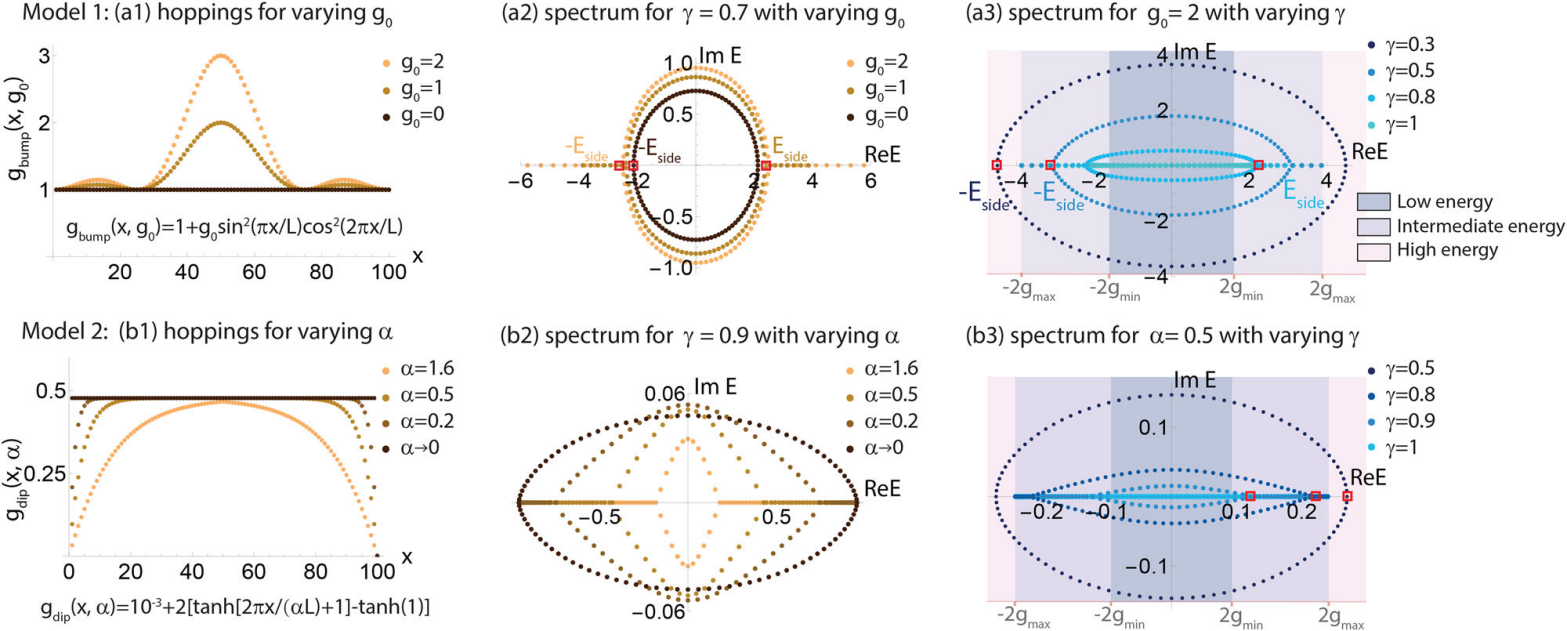
How the spatial profile *g*(*x*) and asymmetry γ of the non‐Hermitian hoppings affect their energy spectra. Shown are two illustrative example models with hopping profiles a1‐a3) *g*(*x*) = *g*_bump_(*x*, *g*_0_) = 1 + *g*_0_sin ^2^(π*x*/*L*)cos ^2^(2π*x*/*L*) and b1‐b3) $g\left(x\right)=g_{\text{dip}}\left(x,\alpha \right)=10^{-3}+2\left(\tanh\left(\frac{2\pi x}{\alpha L}+1\right)-\tanh\left(1\right)\right)$ for *x* ⩽ *L*/2 and symmetric *g*(*x*) about $x=\frac{L}{2}$ for *x* > *L*/2, where *g*_0_ and α respectively control the extent of spatial inhomogeneity. a1,b1) *g*(*x*) profiles for these two models. a2,b2) The corresponding energy spectra at fixed γ, which branch out into real spectral “tails” at branch points ±*E*_side_ for sufficiently strong inhomogeneity *g*_0_ or α. a3,b3) Their corresponding energy spectra at fixed *g*_0_ or α. As the non‐Hermiticity departs from the Hermitian (γ = 1) limit where *E*_side_ = 2*g*_min_, the spectral loops expands and eventually engulfs the real “tails” when *E*_side_ exceeds 2*g*_max_. As elaborated in the text, real tails can only exist in the so‐called intermediate energy regime where 2*g*_min_ < |Re *E*| ⩽ 2*g*_max_.

**Figure 3:**
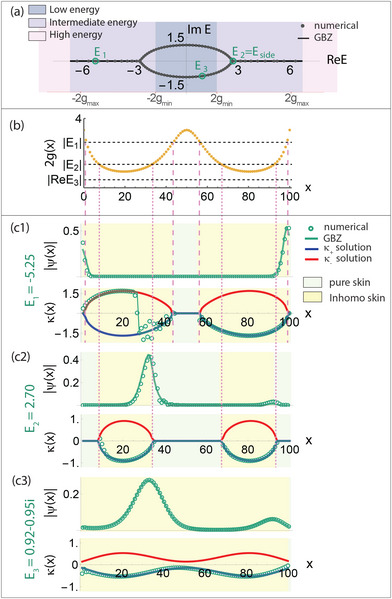
GBZ bifurcations and jumps in the inhomogeneous skin regions. a) Numerically obtained PBC energy spectrum of an illustrative model with *g*(*x*) = 1/(sin (2π*x*/*L*)cos (π*x*/*L*) + 0.3), *L* = 100 and γ = 0.7, which satisfies Equation (20). The branch points ±*E*_side_ in the intermediate energy regime connect the complex spectral segments with the real “tails”, whose existence is constrained by Equation (26). Here, no states exist in the high‐energy regime. b) The doubled spatial hopping profile 2*g*(*x*) is shown against three illustrative chosen eigenenergy values, which determine the pure and inhomogeneous skin regions for their respective eigenstates ψ and GBZs κ(*x*) shown below. c1–c3) The GBZ bifurcates into two branches ±κ_*n*_(*x*) = σ(*x*)Re(cosh ^−1^(*E*_*n*_/2*g*(*x*))) ≠ 0 at inhomogeneous skin regions (pale yellow) where the hoppings are locally weak, i.e., Re(*E*_*n*_) > 2*g*(*x*) or Im(*E*_*n*_) ≠ 0. The GBZ branch chosen by the numerical eigenstates (green), as computed from Equation (16), can jump abruptly in the inhomogeneous skin region (κ(*x*) ≠ 0), as for *E*_1_. The jump position *x*_jump_ = 27 is consistent with Equation (25). However, no jump may occur even if GBZ bifurcation occurs, as for *E*_2_ = *E*_side_ where the numerically‐obtained GBZ adheres to one GBZ solution throughout. No jump can possibly occur when no pure skin region (pale yellow) exists and the GBZ solutions never get to meet, as for *E*_3_ not real in the low energy regime.

In particular, Equation (20) allows the spectrum *E*_*n*_ to be mapped out once σ(*x*) is determined, as will be explained in the following pages. By restricting the 2D complex energy plane to satisfy the constraint equation, the spectrum takes the form of 1D curves or branches, as will be demonstrated later in **Figures** 2, 3.

As a corollary to Equation (20), it is not possible for an entire non‐Hermitian system to consist only of pure skin regions (with κ_*n*_(*x*) = 0), since log γ ≠ 0. This is just another way of saying that any net exponential NHSE accumulation in a periodic system must be smoothed out and compensated in a *g*(*x*)‐dependent way, as given by Equations (14) and (15).

### 2.3 Anatomy of Non‐Hermitian Spectra and Eigenstates for Spatially Inhomogeneous Hoppings between Monoatomic Unit Cells

Figure 2 presents two illustrative models of how the non‐Hermitian spectrum behaves as the hopping asymmetry γ and spatial inhomogeneity are varied. They are *g*(*x*) = *g*_bump_(*x*, *g*_0_) and *g*(*x*) = *g*_dip_(*x*, α), as shown in Figure 2 a1, b1. In the first model, *g*_0_ controls the height of the bump at *x* = *L*/2 (Figure 2 a1). In the second model, α controls the depth of the dip in the hopping amplitude near the ends *x* = 1, *L* (Figure 2 b1).

Even though both hopping profiles result in rather different spectra, they universally behave in qualitatively similar ways (Figure 2 a2, b2). In both cases, the spectrum initially assumes the form of the Hatano‐Nelson PBC spectral ellipse [Equation (17)] (black) when there is no spatial hopping inhomogeneity, i.e., *g*_0_ = 0 or α = 0. However, when the spatial inhomogeneity *g*_0_ or α is introduced, it generically deforms the spectral loop and saliently introduces real spectral branches or “tails” at its sides. We call the eigenenergy branch points where the branches join the loop as ±*E*_side_ [red square markers in (a2) and (a3)]. These real spectral branches exist only when the non‐Hermiticity is not excessively strong, emerging in Figure 2 a3,b3 as the hopping asymmetry γ decreases toward the Hermitian limit (γ = 1). As will be proven later in this section, these branches can only exist for 2*g*_min_ < |Re(*E*)| ⩽ 2*g*_max_ (dubbed the intermediate energy regime), implying that they can only appear when the spectral loop is contained within |Re(*E*)| < 2*g*_max_.

Since directed amplification is supposed to continue indefinitely around a homogeneous PBC loop, the emergence of these real eigenenergy branches indicates that spatial inhomogeneity can locally obstruct this amplification, effectively segmenting the system's spectral response. For *g*_dip_(*x*, α) (Figure 2 b2), it is also interesting that Max(Im(*E*)) peaks slightly at moderate values of α ≈ 0.2, indicative of a slight enhancement of NHSE feedback gain due to hopping inhomogeneity, even though nonzero α corresponds to regions of weak hoppings that should have reduced the overall hopping energies.

#### 2.3.1 Real Spectral Branches from Generalized Brillouin Zone (GBZ) Discontinuities

To derive the real eigenenergy tail segments that would appear given a generic *g*(*x*) and γ ≠ 1, we turn to the boundary condition (Equation (20)), which constrains the set of possible *E*_*n*_ eigensolutions for a given GBZ choice function σ(*x*). Since the full solution set of Equation (20) consists of 1D spectral curves in the complex energy plane, additionally restricting to the real line (Im(*E*) = 0) reduces the solutions to one or more isolated points. In particular, for constant σ(*x*) = −Sgn[log γ], i.e., continuous phase‐space GBZs, the real eigenenergies are found to be *E*_*n*_ = ±*E*_side_, as defined by

(21) $$\begin{array}{c} \;\text{Re}\sum_{x=1}^{L}\cosh^{-1}\left(\frac{E_{\text{side}}}{2g(x)}\right)=L\left|\log\gamma \right| \end{array}$$

To show that ±*E*_side_ indeed bounds the two sides of a complex spectral loop [Figure 2 a2,a3,b2,b3], we invoke the following relations between a generic eigenenergy *E*_*n*_ and its *k*_*n*_(*x*) and κ_*n*_(*x*) [Equation (11)]

(22) $$\begin{array}{cc} & \frac{\text{Re}(E_{n})}{2g(x)}=\cos\left(k_{n}\left(x\right)\right)\cosh\left(\kappa_{n}\left(x\right)\right) \end{array}$$

(23) $$\begin{array}{cc} & \frac{\text{Im}(E_{n})}{2g(x)}=-\sin\left(k_{n}\left(x\right)\right)\sinh\left(\kappa_{n}\left(x\right)\right) \end{array}$$

which can be obtained by separating the real and imaginary parts of Equation (9). Since κ_*n*_(*x*) cannot identically vanish due to the boundary condition [Equation (20)], sin (*k*_*n*_(*x*)) must vanish identically for the real eigenenergy *E*_side_ [Equation (23)]. To continue satisfying Equation (20) for Re(*E*) < *E*_side_, *k*_*n*_(*x*) in Equation (22) can simply be tuned up; however, doing so inevitably also introduces non‐zero Im(*E*), as required by Equation (23). As such, for Re(*E*) < *E*_side_, *k*_*n*_(*x*) generically generates continuous spectral curves extending into the complex plane.

Most interestingly, if the GBZ choice function σ(*x*) were to vary with *x*, exhibiting jumps between ±1 values, it is possible to realize a whole continuum of real eigenenergies *E*_*n*_, i.e., the real spectral “tails” that all satisfy Equation (20), as illustrated in Figure 3a. Previously, with constant σ(*x*), real energies with *E*_*n*_ > *E*_side_ cannot satisfy Equation (20) because the inverse cosh function is monotonically increasing, such that their Re(cosh ^−1^(*E*_*n*_/2*g*(*x*))) contributions must exceed that from *E*_side_. However, if σ(*x*) is non‐uniform (i.e., exhibits phase‐space GBZ discontinuities), jumping at *x* = 1 and *x* = *x*_jump_

(24) $$\begin{array}{c} \;\sigma \left(x\right)=\left\{\begin{array}{cc} 1 & 1\leq x<x_{\text{jump}}\\ -1 & x_{\text{jump}}\leq x\leq L \end{array}\right.\; \end{array}$$

the constraint Equation (20) becomes

(25) $$\begin{array}{c} \left(\sum_{x=x_{\text{jump}}}^{L}-\sum_{x=1}^{x_{\text{jump}}-1}\right)\text{Re}\left(\cosh^{-1}\left(\frac{E_{n}}{2g(x)}\right)\right)=L\left|\log\gamma \right| \end{array}$$

which can be satisfied by a continuum of real *E*_*n*_ > *E*_side_ as the GBZ discontinuity position *x*_jump_ is decreased continuously from *L*. As shown for an illustrative *g*(*x*) (Figure 3b), such discontinuities are indeed numerically observed (Figure 3 c1) in a typical real‐energy state whose absolute energy lies between |*E*_side_| and |2*g*_max_|.

In the above, we have established that, due to the freedom in toggling between two different GBZ solutions ±κ_*n*_(*x*) in a position‐dependent manner, as encoded by σ(*x*) jumps, extensively many real energy eigenstates can exist in a PBC system with spatially inhomogeneous hopping amplitudes. Its extensive nature arises from the spatially extensive pockets of weak *g*(*x*) hoppings, which behave like defects admitting many “frozen” eigenmodes. This is of great physical significance since these real energy states do not grow with time, unlike almost every eigenstate in a clean PBC NHSE system,^[^ ^70^ ^]^ which has complex energy due to unfettered directional amplification. The juxtaposition of two different GBZs κ_*n*_(*x*) at a spatial GBZ discontinuity *x* = *x*_jump_ effectively gives rise to an effective spatial “barrier” that curtails directional state growth, at least for eigenstates lying in the real spectral branches. Alternatively, this can also be qualitatively understood as a generalization of a critical skin effect system, whose pair of competing weakly‐coupled OBC NHSE chains behaves like a single PBC ring with two localized regions of weak *g*(*x*). This work reveals unique advantages of spatially inhomogeneous non‐Hermitian systems with GBZ jumps for sensitivity control. Unlike conventional NHSE systems, our model supports real‐energy modes without relying on open boundaries and enables spatially tunable response regions through GBZ branch discontinuities. The resulting enhancement in spectral density near specific energy ranges allows for stronger responses to weak perturbations, offering promising potential for high‐precision and spatially selective sensing applications.

#### 2.3.2 Spectral Behavior in Low, High, and Intermediate Energy Regimes

Having discussed how the hopping inhomogeneity leads to the real spectral segments, here we discuss how it affects the full non‐Hermitian (γ ≠ 1) spectral behavior in the whole complex plane. To showcase the universality of our arguments, we introduce an additional model with a spatially inhomogeneous hopping profile *g*(*x*) that contains a smooth bump at *x* = *L*/2 and a sharp bump at *x* = 1 or *L* (Figure 3).

Below, we classify the eigenenergies *E*_*n*_ into three regimes by comparing Re(*E*_*n*_) against the lower and upper bounds of *g*(*x*), as colored in Figure 2 a3,b3 and Figure 3a:

- **Low energy regime with |Re(*E*_*n*_)| ⩽ 2*g*_min_**: Only complex *E*_*n*_ allowed, with κ_*n*_(*x*) ≠ 0 (inhomogeneous skin) across all *x*.
  To see why the eigenenergies *E*_*n*_ must be complex, first note that *k*_*n*_(*x*) ≠ 0, because |Re(*E*_*n*_)/2*g*(*x*)| ⩽ 1 in the LHS of Equation (22), but |cosh (κ_*n*_(*x*))| ⩾ 1 on the RHS. This is the only possibility because the special case |Re(*E*_*n*_)/2*g*_min_(*x*)| = 1 = |cosh (κ_*n*_(*x*))| with |Re(*E*_*n*_)| = 2*g*_min_(*x*) cannot hold, as κ_*n*_(*x*) cannot identically vanish due to the PBC condition [Equation (20)]. Equation (23) then forces Im(*E*_*n*_) to be nonzero.
  Note that this low energy regime also exists even when *g*(*x*) is spatially uniform, since the above arguments do not involve the details of the *g*(*x*) profile, only that |Re(*E*_*n*_)| < 2*g*_min_. This is already evident from Figure 2 a2, where the low energies form the top of the spectral loops for any *g*(*x*) profile. In particular, similar to the spatially homogeneous case, the phase‐space GBZ is also continuous with κ_*n*_(*x*) ≠ 0 everywhere (Figure 3 c3), as required for complex *E*_*n*_ with *k*_*n*_(*x*) ≠ 0 [Equation (23)].
- **High energy regime with |Re(*E*_*n*_)| > 2*g*_max_**: Either no states at all, or two branches of complex *E*_*n*_ with κ_*n*_(*x*) ≠ 0 (inhomogeneous skin) meeting at an isolated real eigenenergy point *E*_side_.
  These two possibilities correspond to γ > 0.3 (γ = 0.3) in Figure 2 a3 and γ > 0.5 (γ = 0.5) in Figure 2 b3. The argument partially mirrors that of the low‐energy regime. Here, κ_*n*_(*x*) ≠ 0 because |Re(*E*_*n*_)/2*g*(*x*)| > 1 in the LHS of Equation (22), but $|\;\cos(k_{n}\left(x\right))|\leq 1$ on the RHS. With κ_*n*_(*x*) ≠ 0 for all *x*, Equation (23) forces Im(*E*_*n*_) to also not disappear, except for the possible special real solution, which we call *E*_*n*_ = *E*_side_, where *k*_*n*_(*x*) = 0 for all *x*.
- **Intermediate energy regime with 2*g*_min_ < |Re(*E*_*n*_)| ⩽ 2*g*_max_**:Most interesting is that this intermediate energy regime is characterized by *E*_*n*_ lying between the lower and upper bounds 2(*g*_min_, *g*_max_] of the hopping energy *g*(*x*) (Figures 2 a3,b3 and 3a), which has no analogue in the spatially homogeneous limit (where *g*_min_ = *g*_max_). This regime is most intricate because, for a fixed value of *E*_*n*_, there simultaneously exist spatial intervals with Re *E*_*n*_ > 2*g*(*x*) as well as Re *E*_*n*_ ⩽ 2*g*(*x*), giving rise to coexisting locally high and low energy regions where κ_*n*_(*x*) = 0 and κ_*n*_(*x*) ≠ 0, respectively.
  In particular, it is only in this intermediate energy regime that a continuum of real *E*_*n*_ can exist. As shown in Figure 3a,b, if 2*g*_min_ < *E*_side_ ⩽ 2*g*_max_, the two complex spectral branches from the low‐energy regime would meet at *E*_*n*_ = *E*_side_ and continue as a real energy tail that extends till *E*_*n*_ = 2*g*_max_, the upper limit of the intermediate energy regime. These real energy segments align with the predicted GBZ discontinuities [Equation (25)], confirming the scenario discussed earlier.
  To understand the role of *E*_side_, note that it is the “extremal” solution of the boundary equation [Equation (20)] that still keeps σ(*x*) constant, i.e., $\log\left(\gamma \right)=\frac{1}{L}\sum_{x=1}^{L}\kappa_{\text{side}}\left(x\right)$. For other real energies *E*_*n*_ with |*E*_*n*_| > *E*_side_, the constraint |κ_*n*_| > |κ_side_| would then require position‐dependent σ(*x*) such as to satisfy the boundary equation [Equation (20)]. The upper limit of this continuum of real energies is given by *E*_*n*_ = 2*g*_max_, since cosh κ_*n*_(*x*) ⩽ 1 and sin *k*_*n*_(*x*) = 0 in Equations (22) and (23).
  As shown in Figure 3a,b, if 2*g*_min_ < *E*_side_ ⩽ 2*g*_max_, the two complex spectral branches from the low‐energy regime would meet at *E*_*n*_ = *E*_side_ and continue as a real energy tail that extends until *E*_*n*_ = 2*g*_max_, the upper limit of the intermediate energy regime. These real energies in the tail correspond to phase‐space GBZ discontinuities at *x* = *x*_jump_, as given in Equation (25), although non‐zero contributions to the sum only come from κ_*n*_(*x*) ≠ 0 (inhomogeneous skin) regions where *g*(*x*) < *E*_*n*_/2. As shown in Figure 3 c1,c2 for eigensolutions *E*_1_, *E*_2_ that lie in the intermediate energy regime [Figure 3a purple], the jump/s can be numerically extracted from the spatial wavefunction profile via Equation (16). In Figure 3 c1, the numerical fit to either ±κ(*x*) solution is good, except at the rather abrupt jump. In Figure 3 c2, there is no jump as the numerical wavefunction adheres to only one GBZ solution branch throughout; in Figure 3 c3, no jump is possible because the two κ(*x*) solutions do not even touch.
  However, if *E*_side_ exists in the high‐energy regime, i.e., *E*_side_ > 2*g*_max_ or
(26) $$\frac{1}{L}\sum_{x=1}^{L}\cosh^{-1}\left(g_{\max}/g\left(x\right)\right)<\left|\log\gamma \right|$$
  there will be no real spectral tail, and the complex spectral branches simply meet at the real point *E*_side_ and terminate there (Figure 2 a3, 2 b3). This would definitely be the case when *g*(*x*) is uniform, since *g*(*x*) = *g*_max_. Hence Equation (26) gives the threshold for the absence of real eigenenergies: as the hopping asymmetry log γ is increased, directed amplification becomes stronger, and greater spatial inhomogeneity *g*(*x*) in the hoppings is needed to stop the amplification and produce asymptotically dynamically stable eigensolutions.

Our theoretical framework, though developed for smoothly varying inhomogeneities, can be naturally extended to quasi‐disordered or piecewise‐continuous systems, where the local GBZ structure and the associated skin‐depth profile κ(*x*) remain well‐defined over mesoscopic regions. In such scenarios, the key physical phenomena identified here—such as real spectral branches and spatially displaced non‐Bloch localization—are expected to persist in a locally modulated form. Even in more strongly disordered regimes, where *g*(*x*) fluctuates rapidly and the GBZ factor β_*n*_(*x*) may become irregular or non‐differentiable, the phase‐space formalism still offers a valuable perspective on spectral fragmentation and non‐Hermitian localization. These insights extend the relevance of our results beyond idealized modulations, suggesting applicability to a broad range of physical platforms with engineered or intrinsic disorder.

### 2.4 Phase‐Space Generalized Brillouin Zone (GBZ) for Inhomogeneous Two‐Component Chains

Here, we generalize previous results on monoatomic unit cells to spatially inhomogeneous lattices with diatomic unit cells, such that the odd and even hoppings have independent spatial profiles *g*_1_(*x*) and *g*_2_(*x*). Having a non‐trivial unit cell leads to the appearance of multiple spectral bands that not only enrich the phase‐space GBZ and complex spectral graphs,^[^ ^71^, ^72^ ^]^ but also host special topological “soft‐interface” zero modes that have no analogue in spatially homogeneous PBC or OBC systems.

**Figure 4:**
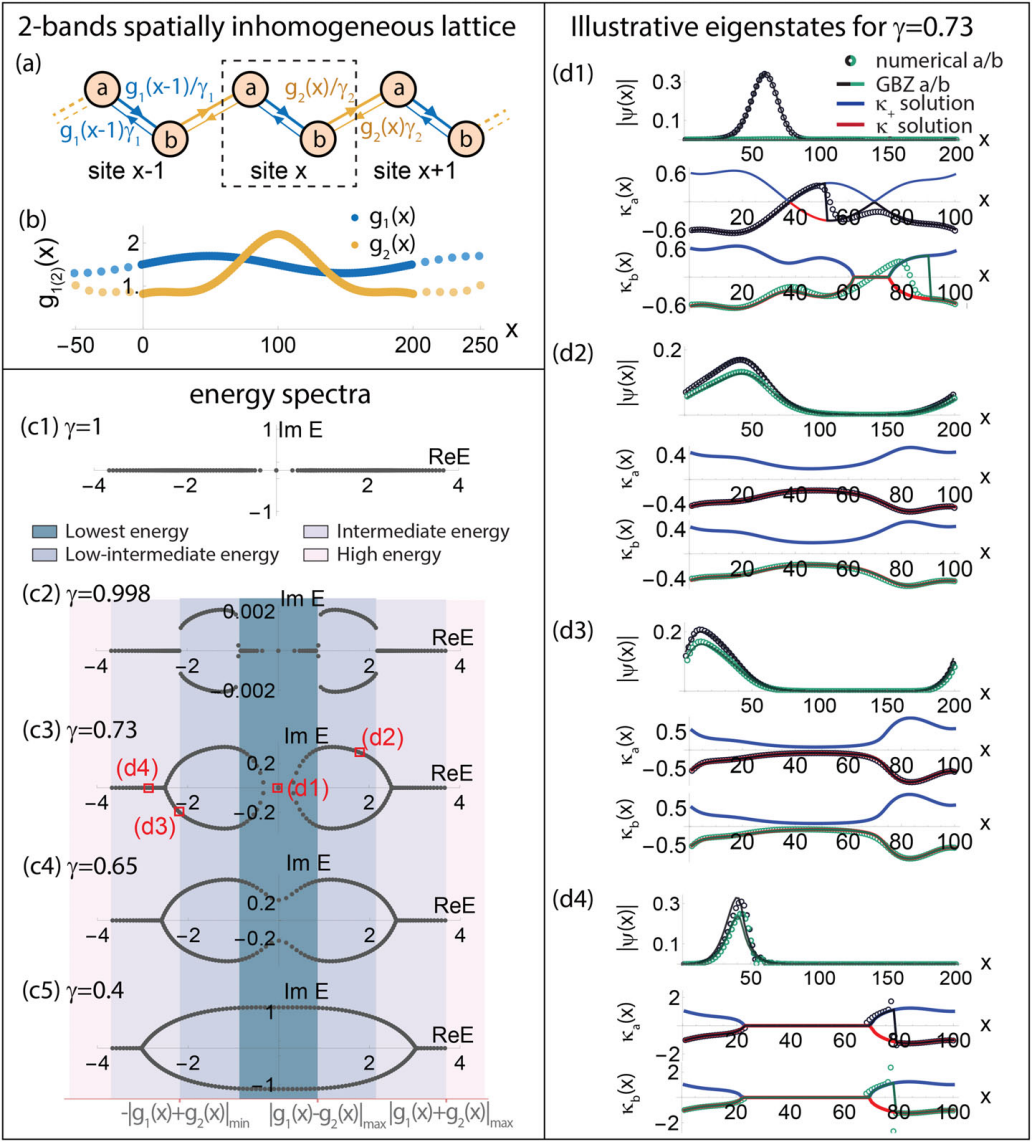
The energy regimes and bifurcated GBZs of 2‐component spatially inhomogeneous non‐Hermitian lattices. a) Schematic of our spatially inhomogeneous PBC lattice with two atoms per unit cell (dashed), such that the inhomogeneous intra‐cell hoppings and inter‐cell hoppings have independent profiles *g*_1_(*x*) and *g*_2_(*x*). b) The illustrative profiles used, with *g*_1_(*x*) = 1.5 + 0.2sin (2π*x*/*L*) and *g*_2_(*x*) = (0.001|(*x* − *L*/2)^1.8^| + 0.5)^−1^ + 0.2cos (4π*x*/*L*) + 0.01, with *L* = 200. c1–c5) Energy spectra as the combined hopping asymmetry γ = γ_1_γ_2_ is tuned from the Hermitian γ = 1 limit to γ = 0.4. Different from the one‐component case, there are four distinct energy regimes and two spectral loops that terminate at real “tails” in the lowest and intermediate energy regimes. A spectral transition occurs as γ is lowered from γ = 0.73 to 0.65, when the two complex loops coalesce into one, destroying the zero mode. d1–d4) Wavefunctions of four representative numerical eigenstates (black/green for component *a*/*b*) from (c3), and their approximations by the phase‐space GBZs ±κ_*a*_(*x*), ±κ_*b*_(*x*). Analogous to one‐component cases, states on the real “tails” possess discontinuous phase‐space GBZs and exhibit *x*_*j*, jump_ jumps such as to satisfy the PBC constraint Equation (33). Interestingly, the zero mode of (d1) exhibits completely distinct κ_*a*, *b*_(*x*) profiles both analytically and numerically, a spectacular consequence of the spatial inhomogeneity of the hoppings.

A 1D two‐component (diatomic unit cell) lattice with spatially inhomogeneous hoppings is defined by

(27) $$\begin{array}{cc} H_{\text{2-comp}} & =\sum_{x=1}^{L}g_{1}\left(x\right)\left(\frac{1}{\gamma_{1}}\left|x,a\right\rangle \left\langle x,b\right|+\gamma_{1}\left|x,b\right\rangle \left\langle x,a\right|\right)\\ & \;+\sum_{x=1}^{L-1}g_{2}\left(x\right)\left(\frac{1}{\gamma_{2}}\left|x,b\right\rangle \left\langle x+1,a\right|+\gamma_{2}\left|x+1,a\right\rangle \left\langle x,b\right|\right)\\ & \;+g_{2}\left(L\right)\left(\frac{1}{\gamma_{2}}\left|L,b\right\rangle \left\langle 1,a\right|+\gamma_{2}\left|1,b\right\rangle \left\langle L,a\right|\right) \end{array}$$

as illustrated in **Figure** **4**a, generalizing the well‐known (spatially uniform) SSH model. Each diatomic unit cell is indexed by its position *x*, and hoppings across atoms *a*, *b* within each unit cell have amplitudes *g*_1_(*x*)γ_1_ and *g*_1_(*x*)/γ_1_ in either direction, where γ_1_ is the intra‐cell hopping asymmetry. Likewise, hoppings connecting atoms *b*, *a* across adjacent unit cells have amplitudes *g*_2_(*x*)γ_2_ and *g*_2_(*x*)/γ_2_ in either direction.

We denote the *n*‐th eigenstate as $\Psi_{n}=\sum_{x}\sum_{j=a,b}\psi_{n,j}\left(x\right)\left|x,j\right\rangle$. Inspired by the single‐component ansatz [Equation (3)], we write

(28) $$\begin{array}{c} \psi_{n,j}\left(x\right)=\frac{{\left(\gamma_{1}\gamma_{2}\right)}^{x}}{\sqrt{g_{1}\left(x\right)g_{2}\left(x\right)}}\prod_{x^{\prime }=1}^{x}\beta_{n,j}\left(x^{\prime }\right) \end{array}$$

Assuming smooth spatial profiles and adiabatic continuity [See Section Detailed Derivation for the Two‐Component Phase‐Space GBZ], the local GBZ factor β_*n*, *j*_(*x*) satisfies the same functional equation as in the single‐component case [Equation (8)]

(29) $$\begin{array}{c} \frac{1}{2}\left(\beta_{n,j}\left(x\right)+\frac{1}{\beta_{n,j}\left(x\right)}\right)=\omega_{j}\left(E_{n},x\right) \end{array}$$

with

(30) $$\begin{array}{cc} \omega_{a} & =\frac{E_{n}^{2}-g_{1}^{2}\left(x\right)-g_{2}^{2}\left(x-1\right)}{2\sqrt{g_{1}\left(x-1\right)g_{1}\left(x\right)g_{2}\left(x-1\right)g_{2}\left(x\right)}}\\ \omega_{b} & =\frac{E_{n}^{2}-g_{1}^{2}\left(x\right)-g_{2}^{2}\left(x\right)}{2\sqrt{g_{1}\left(x\right)g_{1}\left(x+1\right)g_{2}\left(x-1\right)g_{2}\left(x\right)}} \end{array}$$

The GBZ branches are thus given by

(31) $$\begin{array}{c} \beta_{n,j,\pm }\left(x\right)=\exp\left(\pm \cosh^{-1}\omega_{j}\left(E_{n},x\right)\right) \end{array}$$

from which the inverse skin depth follows

(32) $$\begin{array}{c} \kappa_{n,j,\pm }\left(x\right)=\mp \text{Re}\left[\cosh^{-1}\omega_{j}\left(E_{n},x\right)\right] \end{array}$$

To determine the eigenenergy spectrum and enforce PBC constraints, we introduce branch selector functions σ_*j*_(*x*) = ±1 and generalize Equation (20) to

(33) $$\begin{array}{c} \log\left(\gamma_{1}\gamma_{2}\right)=-\frac{1}{L}\sum_{x=1}^{L}\sigma_{j}\left(x\right)\;\text{Re}\left(\cosh^{-1}\omega_{j}\left(E_{n},x\right)\right) \end{array}$$

Hence, the spatial profile of each component is

(34) $$\begin{array}{c} |\psi_{n,j}\left(x\right)|=\frac{{\left(\gamma_{1}\gamma_{2}\right)}^{x}}{\sqrt{g_{1}\left(x\right)g_{2}\left(x\right)}}\prod_{x^{\prime }=1}^{x}\exp\left(-\kappa_{n,j}\left(x^{\prime }\right)\right) \end{array}$$

where κ_*n*, *j*_(*x*) = −σ_*j*_(*x*) Re(cosh ^−1^ω_*j*_(*E*_*n*_, *x*)).

Notably, κ_*n*, *a*_(*x*) and κ_*n*, *b*_(*x*) can differ substantially in certain regimes, particularly near topological transition points where soft interface modes appear (see Section 2.5). The jump structure of σ_*j*_(*x*) continues to encode phase‐space GBZ bifurcations, with associated spectral and topological implications.

#### 2.4.1 Real Spectra and Phase‐Space Generalized Brillouin Zone (GBZ) Discontinuities for Two‐Component Chains

On first impression, the two‐component spatially inhomogeneous problem may seem no more complicated than the one‐component case, since its spectrum can be obtained by replacing *E*_*n*_/2*g*(*x*) with ω_*j*_(*E*_*n*_, *x*), *j* = *a*, *b* (Figure 4). However, since ω_*j*_(*E*_*n*_, *x*) has a more complicated spatial dependence, it, in fact, sets more sophisticated and subtle constraints on the spectrum.

Previously, it was established that a real energy continuum requires the presence of phase‐space GBZ discontinuities *x*_jump_ [Equation (25)], which occur when |*E*_*n*_/2*g*(*x*)| ⩽ 1 for some but not all *x*, i.e., 2*g*_min_ ⩽ Re(*E*_*n*_) ⩽ 2*g*_max_ (the intermediate energy regime). For this two‐component case, a real spectral continuum thus requires that |ω_*j*_(*E*_*n*_, *x*)| ⩽ 1 for some but not all *x*, such that Equation (33) can be satisfied for a continuum of real *E*_*n*_ by continuously adjusting *x*_jump_ in σ_*j*_(*x*). Substituting this condition into Equation (30) and assuming sufficiently smooth spatial inhomogeneities such that *g*_1, 2_(*x*) ≈ *g*_1, 2_(*x* ± 1),

(35) $$\begin{array}{cc} |g_{1}\left(x\right)+g_{2}{\left(x\right)|}_{\min} & <|\text{Re}\left(E_{n}\right)\left|<\right|g_{1}\left(x\right)+g_{2}\left(x\right){|}_{\max} \end{array}$$

(36) $$\begin{array}{cc} \text{or}\;\;0 & <|\text{Re}\left(E_{n}\right)\left|\leq \right|g_{1}\left(x\right)-g_{2}\left(x\right){|}_{\max} \end{array}$$

Unlike the one‐component case where *g*_1_(*x*) = *g*_2_(*x*) = *g*(*x*), real energies can also exist within an additional low(est) energy regime 0 < |Re(*E*_*n*_)| < |*g*_1_(*x*) − *g*_2_(*x*)|_max_ [Equation (36)], where |Re(*E*_*n*_)| is not larger than the hopping amplitude difference between the odd and even bonds. This new regime obviously does not exist in the one‐component case where Equation (35) simply reduces to the intermediate energy regime. As such, the spectral plane is divided into up to four different energy regimes:

- **Lowest energy regime with |Re(*E*_*n*_)| < |*g*_1_(*x*) − *g*_2_(*x*)|_max_**: Real energies are possible.
- **Low‐intermediate energy regime with |*g*_1_(*x*) − *g*_2_(*x*)|_max_ ⩽ |Re(*E*_*n*_)| ⩽ |*g*_1_(*x*) + *g*_2_(*x*)|_min_**: Only complex energies are possible; however, this regime may not exist for sufficiently dissimilar *g*_1_(*x*), *g*_2_(*x*).
- **Intermediate energy regime with |*g*_1_(*x*) + *g*_2_(*x*)|_min_ < |Re(*E*_*n*_)| ⩽ |*g*_1_(*x*) + *g*_2_(*x*)|_max_**: Real energies are possible.
- **High energy regime with |*g*_1_(*x*) + *g*_2_(*x*)|_max_ < |Re(*E*_*n*_)|**: Complex energy branches (if any) join up along the real line.

In particular, continua of real energies can exist in two distinct scenarios: in the intermediate energy regime, which exists only for spatially inhomogeneous systems (similar to the one‐component case), or in the lowest energy regime, where the contrast between adjacent *g*_1_(*x*), *g*_2_(*x*) hoppings is sufficient to block directed amplification.

Shown in Figure 4 is an illustrative two‐component model (Figure 4a) with intra‐ and inter‐component hopping inhomogeneity profiles *g*_1_(*x*), *g*_2_(*x*) that intersect at two different locations (Figure 4b). As non‐Hermiticity is introduced and γ departs from unity (Figure 4c), spectral loops appear across the lowest to intermediate energy regimes (cyan to light purple). Even though real energies are allowed in the lowest energy regime (cyan), the complex loops join up to form one loop with sufficiently strong non‐Hermitian asymmetry γ, a phenomenon with no single‐component analog.

It is instructive to examine the GBZ profiles of the representative eigenstates indicated in Figure 4 c3 by red hollow squares. As presented in Figure 4d, only the real energy eigenstates (d1) and (d4) possess pure skin regions where at least one GBZ component is degenerate, i.e., κ_*a*_(*x*) = 0 or κ_*b*_(*x*) = 0. These degeneracies allow for “hidden” switching of the actual GBZ branches adopted by the numerical eigenstates ψ, which in turn gives rise to spectacular GBZ jumps, i.e., discontinuities in σ_*j*_(*x*) = ±1 necessary for satisfying Equation (33). While both GBZ components κ_*a*, *b*_(*x*) look identical in (d2)‐(d4), they can become completely distinct when the spatial gradient *g*_2_(*x*) − *g*_2_(*x* − 1) dominates over the eigenenergy *E*_*n*_ in ω_*a*/*b*_(*E* = 0, *x*) of Equation (30), as for the zero mode of (d1).

### 2.5 Topological Transitions from Spatial Inhomogeneity

Most interestingly, spatial hopping inhomogeneity in a two‐component lattice can also drive topological phase transitions. Ordinarily, in a non‐Hermitian SSH model with uniform *g*_1_(*x*) = *g*_1_ and *g*_2_(*x*) = *g*_2_ hopping amplitudes, it is well‐known that a topological phase boundary occurs at “domain walls” where they swap. Here, with spatially inhomogeneous *g*_1_(*x*) and *g*_2_(*x*), it is reasonable to expect that *g*_1_(*x*) = *g*_2_(*x*) intersections still function as topological interfaces, since they demarcate the regions *g*_1_(*x*) < *g*_2_(*x*) and *g*_2_(*x*) > *g*_1_(*x*) that are supposed to represent different phases.

**Figure 5:**
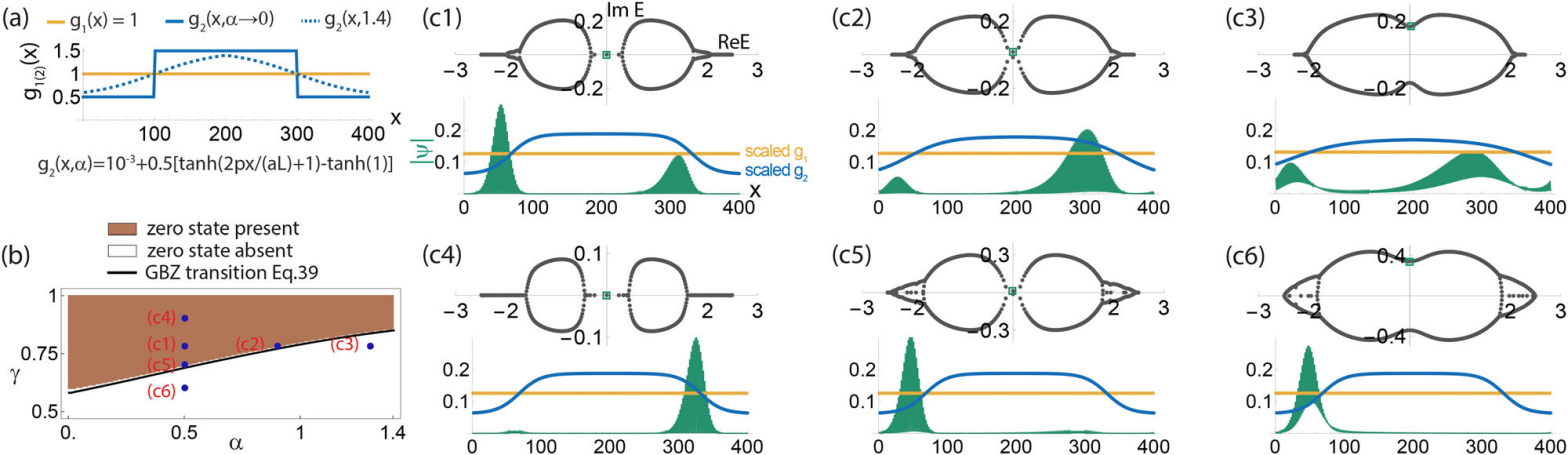
Topological phase transition from varying the strength and smoothness of the spatial hopping profile. a) PBC hopping profile, with uniform intra‐unit cell hoppings *g*_1_(*x*) = 1 and non‐uniform inter‐unit cell hopping strengths $g_{2}\left(x,\alpha \right)=1+0.5\tanh\left(2\pi \frac{x-\frac{L}{4}}{\alpha L}\right)$, $-\frac{L}{2}<x\leq \frac{L}{2}$, with *L* = 200. *g*_1_(*x*) and *g*_2_(*x*) intersect to form domain walls at $x=\frac{L}{4}$ and $\frac{3L}{4}$, with wall steepness diverging to form hard boundaries as α → 0. b) The numerically‐determined region (brown) with robust zero modes (numerical tolerance |*E*| < 0.015) in the parameter space of α and non‐Hermitian asymmetry γ. It is accurately bounded by the transition curve (black) from Equation (38). c1–c6) Spectra and corresponding zero mode (or minimal |*E*_*n*_| state) [Equation (34)] at illustrative parameter values given in (b). Topological zero modes always occupy only one sublattice (dashed or solid green) per unit cell at the same *x*, just like familiar SSH zero modes, and accumulate against the domain wall intersections *g*_1_(*x*) = *g*_2_(*x*).

**Figure 6:**
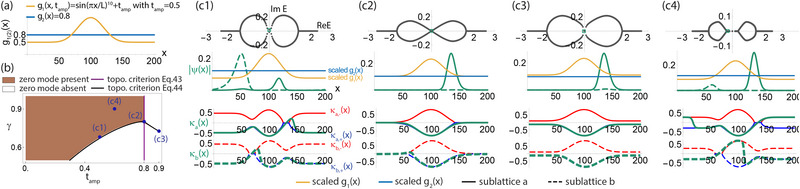
Topological phase transition and the crucial role of GBZ jumps. a) The PBC hopping profile, as given by constant *g*_2_(*x*) and inhomogeneous *g*_1_(*x*, *t*_amp_) = sin (π*x*/*L*)^10^ + *t*_amp_, *L* = 200, with the offset *t*_amp_ controlling whether *g*_1_(*x*) and *g*_2_(*x*) intersect to form topological domain walls. b) The numerically determined region hosting topological zero modes (brown, with numerical tolerance |*E*| < 0.02) in the parameter space of γ and *t*_amp_. It is accurately demarcated by Equation (37) (purple) and Equation (38) (black), which gives the phase‐space GBZ threshold boundaries between continuous and discontinuous GBZs. c1‐c4) Numerically‐obtained spectra, state profiles [Equation (34)] and GBZ occupancies of illustrative *E* = 0 zero modes in (b). Deeper into the topological regime (c1,c4), the zero modes are always localized near the “soft boundary” defined by *g*_1_(*x*) = *g*_2_(*x*) and occupy only one sublattice (dashed or solid green) per unit cell, even though the other sublattice may be occupied away from the soft boundary. However, unlike the bulk zero mode (c3), only the topological states in (c1) and (c4) have discontinuous κ_*j*_(*x*) jumping between two branches of κ_*j*, ±_(*x*).

However, PBC spatial inhomogeneity complicates the stability of zero modes in various ways. First, among two non‐empty regions separated by a domain wall, one of them must already possess non‐trivial bulk topology in a bipartite system. Second, the bulk is now spatially inhomogeneous, such that its GBZ description rightly lives in phase space and not just momentum space. Third, since phase space encompasses position coordinates, the shape of the domain wall itself affects the topology. Indeed, as will be shown in **Figures** **5** and **6**, the topological modes are not fully confined to *g*_1_(*x*) = *g*_2_(*x*) interfaces but, in fact, penetrate nonlocally and nonexponentially into other parts of the system.

#### 2.5.1 Topological Criteria

Here, we describe a new type of topological robustness protected by GBZ bifurcations. Unlike topological modes at open boundaries, which are simply protected by bulk topological invariants, the inhomogeneous PBC isolated zero modes (i.e., in Figure 4 d1) turn out to be crucially protected by GBZ jumps. These jumps facilitate the realization of real spectra (which includes *E*_*n*_ = 0), as discussed in the paragraphs surrounding Equations (25) and (33), and are also further elaborated in Section Phase‐Space GBZ Transitions for a Fixed State.

As such, to have robust isolated zero modes (and not just trivial *E* = 0 crossings), the following two criteria on γ and *g*_1_(*x*), *g*_2_(*x*) must be satisfied:

(37) $$\begin{array}{cc} & |g_{1}\left(x\right)-g_{2}{\left(x\right)|}_{\min}=0 \end{array}$$

(38) $$\begin{array}{cc} \&\; & |\log\left(\gamma \right)|<|\log\left({\tilde{\gamma }}_{\text{topo}}\right)| \end{array}$$

where

(39) $$\begin{array}{cc} & {\tilde{\gamma }}_{\text{topo}}=\exp\left(\frac{1}{L}\sum_{1<|\omega_{j}\left(E=0,x\right)|}\cosh^{-1}\left|\omega_{j}\left(E=0,x\right)\right|\right) \end{array}$$

is the γ threshold that separates continuous and discontinuous GBZ scenarios, with *j* = *a* or *b* yielding negligible differences in the phase diagram (see Section Phase‐Space GBZ Transitions for a Fixed State).

The criterion |*g*_1_(*x*) − *g*_2_(*x*)|_min_ = 0 [Equation (37)] is simply that the intra‐ and inter‐unit cell hopping profiles *g*_1_(*x*) and *g*_2_(*x*) intersect to form spatial “domain walls” that, by construction, have one side with nontrivial bulk topology. Its implications for the bifurcated GBZ are as follows: when Equation (37) is satisfied and *g*_1_(*x*) and *g*_2_(*x*) are sufficiently smooth, κ_*n* = 0, *j*, ±_(*x*) ≈ 0 [Equations (30) and 32] for some *x* and *j* = *a*, *b*. Since κ_*n* = 0, *j*, ±_ ≠ 0 for most other *x*, Equation (37) can hence be understood as the requirement for the simultaneous existence of both inhomogeneous and pure skin regions in the zero eigenmode.

It is also insightful to recast the criterion $|\log\left(\gamma \right)|<|\log\left({\tilde{\gamma }}_{\text{topo}}\right)|$ [Equation (38)] into a much more intuitive approximate form when *g*_1_(*x*), *g*_2_(*x*) are sufficiently smooth. Neglecting spatial gradients in Equation (30),

(40) $$\begin{array}{cc} \kappa_{n,j,\pm }\left(x\right) & =\mp \text{Re}\left(\cosh^{-1}\left|\omega_{j}\left(E=0,x\right)\right|\right)\\ & \approx \mp \text{Sgn}\left[g_{1}\left(x\right)-g_{2}\left(x\right)\right]\log\frac{g_{1}\left(x\right)}{g_{2}\left(x\right)}\; \end{array}$$

which is just the inverse decay length of SSH‐type topological modes without additional skin localization, valid when the inhomogeneities in *g*_1_(*x*), *g*_2_(*x*) contribute only “adiabatically”. Equation (40) simplifies ${\tilde{\gamma }}_{\text{topo}}$ (for ${\tilde{\gamma }}_{\text{topo}}<1$) to

(41) $$\begin{array}{ccc} {\tilde{\gamma }}_{\text{topo}} & = & e^{-\frac{1}{L}\sum_{x=1}^{L}\kappa_{n,j,-}\left(x\right)}\\ & \approx & {\left(\prod_{g_{2}\left(x\right)>g_{1}\left(x\right)}^{L}\frac{g_{2}\left(x\right)}{g_{1}\left(x\right)}\prod_{g_{2}\left(x\right)<g_{1}\left(x\right)}^{L}\frac{g_{1}\left(x\right)}{g_{2}\left(x\right)}\right)}^{\frac{1}{L}}\\ & = & e^{\langle \left|\log\frac{g_{2}\left(x\right)}{g_{1}\left(x\right)}\right|\rangle } \end{array}$$

where the average $\langle \left|\log\frac{g_{2}\left(x\right)}{g_{1}\left(x\right)}\right|\rangle$ is taken over all *x* ∈ [1, *L*]. As such, for sufficiently smooth *g*_1_(*x*), *g*_2_(*x*), the criterion Equation (38) reduces to

(42) $$|\log\gamma |<\langle \left|\log\frac{g_{2}\left(x\right)}{g_{1}\left(x\right)}\right|\rangle$$

which places an upper bound on the non‐Hermitian hopping asymmetry |log γ|, above which it pumps all states across the inhomogeneous PBC chain and destroys the zero modes. This result applies exclusively for “soft spatial boundaries” with no OBC analog: it gives the upper bound for the eigenfunction to be “patched up” to satisfy the PBC condition [Equation (33)] through appropriately placed GBZ jumps in σ_*j*_(*x*). Note that the criterion Equation (38) (or Equation (42)) is trivially satisfied in the Hermitian limit of log γ = 0, in which only the criterion Equation (37) exists.

#### 2.5.2 Illustrative Examples for the Topological Criteria

In Figures 5 and 6, we showcase numerical topological phase diagrams of two illustrative models and how their phase boundaries are accurately determined by the criteria given in Equations (37) and (38). We also present some illustrative eigenstates [Equation (34)] and highlight the characteristic amplitude profiles and GBZ jumps of isolated zero eigenmodes.

Figure 5 presents a spatial hopping profile (Figure 5a) whose topological phase boundary (Figure 5b) is well approximated by Equation (38) alone. It features a uniform *g*_1_(*x*) = 1 (yellow) and a *g*_2_(*x*) (blue), which cuts *g*_1_(*x*) at domain walls $x=\frac{L}{4}$ and $x=\frac{3L}{4}$. The wall steepness is controlled by a parameter α; as α → 0, the steepness diverges, leading to an OBC‐like hard boundary. The phase boundary curve matches excellently with results from numerical diagonalization (brown) and indicates that, as the domain wall becomes softer (with larger α), the zero mode becomes more fragile, being more easily destroyed as γ moves away from the Hermitian limit γ = 1.

Even though all eigenstates (green) are localized to some extent in a spatially inhomogeneous setting, topological zero modes characteristically occupy only one sublattice, as plotted in Figure 5 c1,c4. As the topological line gap closes and forms a point gap [Figure. 5 c2,c3,c5,c6], the eigenstates start to disperse away from the domain walls, and both sublattices assume nonzero occupancy.

In Figure 6, we present a different spatial hopping profile [Figure 6a] such that its topological phase boundary [Figure 6b] is demarcated by both Equations (37) and (38). As the parameter *t*_amp_ increases, the region where *g*_1_(*x*) (yellow) is larger than *g*_2_(*x*) (blue) broadens until it finally occupies the whole system and no domain wall exists. This scenario is exactly demarcated by the purple line, to the right of which |*g*_1_(*x*) − *g*_2_(*x*)|_min_ = 0 [Equation (37)] no longer holds.

That the zero modes are fundamentally protected by GBZ jumps can be seen in Figure 6c, which showcases three illustrative eigenstates on the threshold boundary given by $|\log\gamma |=|\log{\tilde{\gamma }}_{\text{topo}}|$ [Equation (38)]: (c1) within the topological phase, (c2) at the boundary given by Equation (37), (c3) outside the topological phase, as well as a reference (c4) deep within the topological phase. Evidently, numerical GBZ jumps (green) in their GBZ branches of blue and red curves confirm that the topological nature of the *E* = 0 state corresponds to the presence of a discontinuous jump between κ_*j*, ±_(*x*) and the simultaneous presence of both inhomogeneous and pure skin regions.

### 2.6 Discussion

In this work, we have formulated a new theoretical framework for generically treating the interplay of the NHSE and spatial lattice hopping inhomogeneity, as encoded by γ and *g*(*x*), respectively. This is a subtle scenario because the spatially non‐uniform energy scale not only competes with NHSE accumulation through Wannier‐Stark localization but also distorts the skin accumulation and deforms the effective lattice momentum in a position‐dependent manner.

Central to our formalism is the phase‐space generalized Brillouin zone (GBZ), which captures the effective non‐Bloch deformation in both position and momentum space. For any PBC eigensolution *E*_*n*_, the phase‐space GBZ bifurcates into two possible solution branches within regions of relatively weak hoppings 2*g*(*x*) < *E*_*n*_, leading to non‐exponential “inhomogeneous skin” state profiles. Crucially, discontinuous jumps in the adopted GBZ branch give rise to an emergent degree of freedom that results in real “tails” in the energy spectrum. Physically, these real eigensolutions represent states that are prevented from indefinite growth by spatial inhomogeneity.

Two‐component settings encompass new forms of topological robustness as different *g*(*x*) components intersect to form spatial domain walls. The real spectral solutions from GBZ jumps can also exist at very low energies, including the topological zero modes in particular. Unlike the well‐known topological edge modes, these isolated zero modes are protected by the phase‐space GBZ bifurcations, lending their robustness from the emergent freedom in the GBZ jump positions. As shown both theoretically and numerically, such topological phase boundaries can be accurately predicted through our criteria given by Equations (37) and (38).

By generalizing Equations (28) and (30), our phase‐space GBZ framework can be extended to inhomogeneous systems with arbitrarily many components, such that the GBZ of each component depends non‐linearly on the inter‐component hoppings. Additionally, generalizing the spatially inhomogeneous hoppings beyond nearest neighbors replaces Equation (29) with a higher‐degree Laurent polynomial that splits the GBZ solutions into multiple branches. In all, these are expected to generate far more intricate GBZ jumps, leading to many more hidden degrees of freedom that stabilize new emergent spectral branches, some possibly containing topological modes with higher symmetry.^[^ ^73^, ^74^, ^75^, ^76^ ^]^ Further generalization to higher dimensions and multiple interacting boundary conditions could lead to significant new subtleties in the already fragmented GBZ structure, opening up a vast playground for future research into non‐Hermitian localization.

Experimentally, spatially inhomogeneous non‐Hermitian systems are as accessible as their usual uniform lattice counterparts. Non‐Hermitian lattice models have already been realized in electrical circuits,^[^ ^28^, ^67^, ^77^, ^78^, ^79^, ^80^, ^81^, ^82^, ^83^, ^84^, ^85^ ^]^ cold atoms,^[^ ^86^, ^87^, ^88^, ^89^ ^]^ photonics,^[^ ^90^, ^91^, ^92^ ^]^ programmable quantum simulators,^[^ ^93^, ^94^, ^95^, ^96^, ^97^, ^98^, ^99^, ^100^ ^]^ and mechanical/acoustic systems.^[^ ^101^, ^102^, ^103^ ^]^ In most metamaterial platforms, the effective hopping strengths can be spatially tuned in a versatile manner – for instance, the individual components of an electrical circuit array can be tuned at will, with effectively asymmetric couplings simulated using operational amplifiers.^[^ ^77^, ^104^, ^105^ ^]^ The phase space GBZ can be reconstructed from the eigenstate profiles, which can for instance be obtained in electrical circuits through impedance measurements^[^ ^106^, ^107^, ^108^ ^]^ alongside the resonance spectrum.

## 3 Experimental Section

### Limitations on the Phase‐Space Generalized Brillouin Zone (GBZ) Approach

Although the phase‐space GBZ had been shown to be effective in describing the spectra of inhomogeneous single‐ and double‐component systems, local deviations, fluctuations, or discrepancies may still be observed for certain states, as exemplified in Figure 3 c1,c2, particularly near non‐smooth regions of *g*(*x*) or at the transition of κ(*x*) into the pure skin region. In this section, additional examples of phase‐space GBZs in single‐component systems were presented, and the limitations of the phase‐space GBZ method in various finite‐size physical systems were examined.

**Figure 7:**
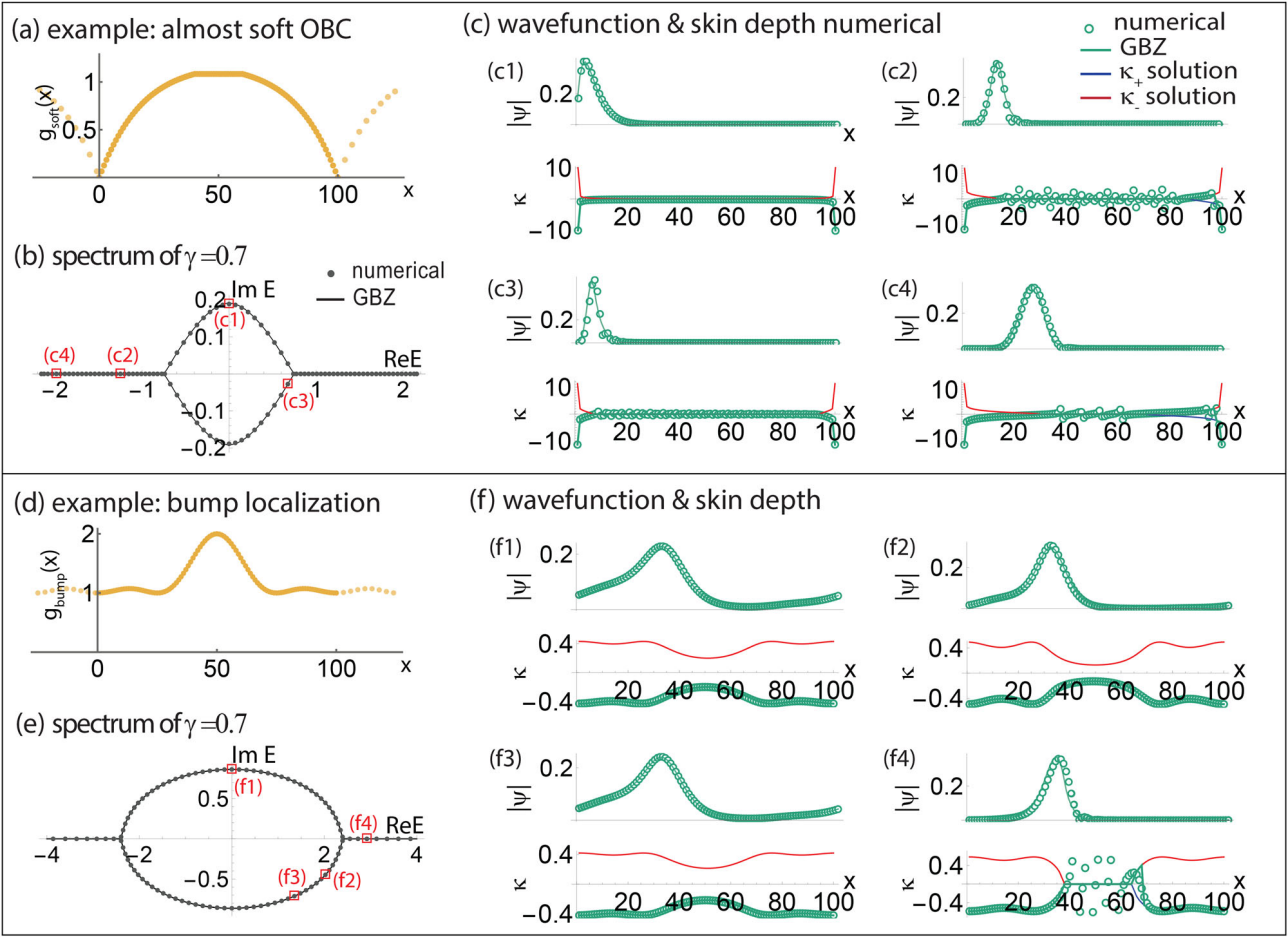
Phase‐space GBZ: further examples. Our phase‐space GBZ approach well‐approximates both energy spectra [Equation (21)] and wavefunctions [Equations (3) and (10)] in smooth 1D single‐band inhomogeneous lattices. Two different inhomogeneous hopping profiles are chosen, *g*(*x*) = *g*_soft_(*x*) a–c) from Equation (44) and *g*(*x*) = *g*_bump_(*x*) d–f) from Equation (44), with γ = 0.7. The numerical results agree almost perfectly with the GBZ solutions, not just in the spectra (b,e), but also the wavefunctions (green) as well as their fits with both continuous and discontinuous GBZ regions (blue and red in c,f). Some fluctuations in the numerically reconstructed GBZ are unavoidable in the pure skin region of finite inhomogeneous systems, as in the discussions in Section 3, but they effectively average to zero in the phase‐space GBZ.

Additional examples of two contrasting inhomogeneous *g*(*x*) hopping profiles and their phase‐space GBZ approximations are first presented in **Figure** **7**, such as to validate the accuracy of our approach. The two illustrative systems are

(43) $$\begin{array}{cc} & g_{\text{soft}}\left(x\right)=\left\{\begin{array}{cc} 5\tanh(\pi \frac{x}{L}+1)+10 & \;\text{for}\;x<\frac{2L}{5}\\ 5\tanh(\pi \frac{2}{5}+1)+10 & \;\text{for}\;\frac{2L}{5}\leq x\leq \frac{3L}{5}\\ 5\tanh(\pi -\frac{\pi x}{L}+1)+10 & \;\text{for}\;x>\frac{3L}{5} \end{array}\right. \end{array}$$

(44) $$\begin{array}{cc} & g_{\text{bump}}\left(x\right)=1+\sin{\left(\pi x/L\right)}^{2}\cos{\left(2\pi x/L\right)}^{2}, \end{array}$$

both with γ = 0.7.

Their full spectra and illustrative states were well approximated by the phase‐space GBZ compared to numerical results (Figure 7b,c,e,f), with minor but observable localized fluctuations: slight deviations appear near the non‐smoothness at *x* = 0 and *x* = *L* in the hopping profile *g*(*x*) = *g*_soft_(*x*) (Figure 7a) as shown in Figure 7 c2; fluctuations also occur in the pure skin regions of states with coexisting pure and inhomogeneous skins (Figure 7c2,c4,f4), where these GBZ κ(*x*) fluctuations lead to localized deviations in the corresponding state profiles. The reasons and the degree of effect of these two sources of inaccuracy will be elaborated later in this section.

*Non‐Smoothness in the Hopping Function*: In introducing the phase‐space GBZ method, sufficiently smooth hopping functions were assumed in Equation (6) such that *g*(*x* ± 1) was approximated as *g*(*x*), leading to the local continuity of the continuous phase‐space GBZ.

**Figure 8:**
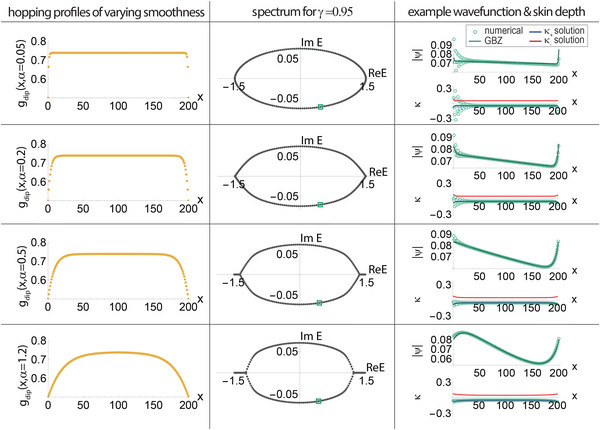
Phase‐space GBZ: effect of discontinuities in the hopping profile *g*(*x*). Numerical reconstruction of the phase‐space GBZ becomes more noisy with increasing local non‐smoothness in *g*(*x*). The cases plotted are based on $g_{\text{dip}}\left(x,\alpha \right)=10^{-3}+\left(\tanh\left(\frac{2\pi x}{\alpha L}+1\right)-\tanh\left(1\right)\right)$ for −*L*/2 < *x* ⩽ *L*/2 [Equation (45)], with varying smoothness parameter α = 0.05, 0.2, 0.5, 1.2 and fixed *L* = 200, γ = 0.95. A small α, corresponding to a sharp discontinuity in *g*(*x*), gives rise to significant Gibbs‐like wavefunction fluctuations and hence fluctuating numerically reconstructed GBZ (green). Increasing α gradually suppresses these fluctuations until a smooth hopping profile and illustrative wavefunction are achieved. The phase‐space GBZ, which is always a smooth curve, does not capture local fluctuations but consistently represents the best averaged trend, providing an optimal smoothed‐fit for the wavefunctions.

While Equation (6) remains valid in the thermodynamic limit *L* → ∞ for any continuous hopping function *g*(*x*) with *g*(*x* + *L*) = *g*(*x*), the approximation precision diminishes for finite *L* due to the local non‐smoothness of *g*(*x*). To illustrate the extent and characteristics of this diminishing accuracy, consider a hopping function with strongly decaying boundary hoppings

(45) $$\begin{array}{cc} g_{\text{dip}}\left(x,\alpha \right) & =\frac{1}{2}-\tanh\left(1\right)\\ & \;+\left\{\begin{array}{cc} \tanh(\frac{2\pi x}{\alpha L}+1) & \;\text{for}\;0<x\leq \frac{L}{2}\\ \tanh(\frac{2\pi (L-x)}{\alpha L}+1) & \;\text{for}\;\frac{L}{2}<x\leq L \end{array}\right. \end{array}$$

as shown in **Figure** **8**. With *L* = 200, the smoothness α was varied from 0.05 to 1.2, and the phase‐space GBZ approximation was compared to numerical results.

With fixed *g*_min_ and *g*_max_, a reduction in α makes the violation of Equation (6) more pronounced, amplifying fluctuations in the wavefunction near the non‐smooth regions of *g*(*x*). Although the phase‐space GBZ assumed perfectly smooth hopping functions and cannot fully capture these fluctuations, it effectively approximates the overall trend of the wavefunction, as demonstrated in Figure 8.

When local fluctuations in the wavefunctions were of interest, it becomes necessary to enhance the smoothness of *g*(*x*), most easily done by just increasing the number of sites within a fixed real‐space length. In the special case of discontinuous *g*(*x*) with sharp jumps over a small range, the phase‐space GBZ could be modified to accommodate sharp boundaries, as discussed later in Section Handling Isolated Hopping Discontinuities.

*Pure Skin Fluctuation in Finite‐Size Systems*:

In the phase‐space GBZ approach, inhomogeneous systems were approximated by assuming the thermodynamic limit *L* → ∞, where the bulk relation becomes fully decoupled between neighboring sites

(46) $$\begin{array}{cc} & \beta_{n}\left(x\right)+\frac{1}{\beta_{n}\left(x\right)}=\frac{E_{n}}{g(x)} \end{array}$$

Within this framework, discontinuous phase‐space GBZ states, which had real eigenenergies and consist of both the pure skin and inhomogeneous skin regions, have κ_*n*, ±_(*x*) that goes from being zero to non‐zero at the boundary between pure skin and inhomogeneous skin regions.

In practice, with finite *L*, neighboring β_*n*_(*x*) does differ slightly, necessitating the use of the original bulk equation [Equation (8)] without the approximation β_*n*_(*x* + 1) ≈ β_*n*_(*x*).

This then brings in problems at the pure‐inhomogeneous skin boundaries, where neighboring phase‐space GBZ factors β_*n*, ±_(*x*) and β_*n*, ±_(*x* + 1) were located within different skin regions, and only one of them is complex. This was mathematically prohibited in Equation (8) for real *E*_*n*_ and *g*(*x*). A necessarily non‐zero imaginary component of β_*n*_(*x*) of pure skin, or equivalently κ_*n*_(*x*) ≠ 0 in the pure skin regions, was required to balance the imaginary equation of Equation (8) in finite‐size systems.

Nevertheless, the phase‐space GBZ solutions κ_*n*, ±_(*x*) = 0 still provide an effective best‐fit approximation for the fluctuating κ_*n*_. Moreover, despite the fluctuations in κ_*n*_(*x*), their impact on the wavefunctions remains minimal, as demonstrated in examples of Figure 7b,c,e,f, where the effect of GBZ fluctuations was restricted locally by the effectively zero skin in the pure skin regions and was compressed by the inhomogeneous skin, which amplifies the wavefunctions at one of the sides.

### Phase‐Space Generalized Brillouin Zone (GBZ) Transitions for a Fixed State

The energy eigenvalue *E*_side_ [Equation (21)] not only bounds the spectral loop on both sides but also served a crucial role in confining the real “tails” within the intermediate energy regime, which was bounded by ±2*g*_max_. In essence, *E*_side_ acted as a transition point between continuous and discontinuous phase‐space GBZ, and was calculated from the boundary requirement Equation (21) (Equation (33)) in single‐component (double‐component) chains, given known non‐Hermiticity γ (γ_1_γ_2_) and hopping function *g*(*x*) (*g*_1, 2_(*x*)).

Here, how one can alternatively investigate the phase‐space GBZ was elaborated by examining how a specific *E*_0_ state undergoes transitions as a certain parameter was varied. For instance, in the topological investigation of the zero‐energy state *E*_0_ = 0, the non‐Hermiticity γ was tuned such that the state undergoes a transition between topologically trivial and non‐trivial phases. The concept of threshold value $\tilde{\gamma }$ of γ, defined for a fixed specified *E*_0_, was hereby proposed. In a single‐component system, it is given by

(47) $$\begin{array}{cc} \tilde{\gamma }\left(E_{0}\right)= & \exp\left(-\frac{1}{L}\sum_{x=1}^{L}\cosh^{-1}\left(\frac{\text{Re}\;E_{0}}{2g(x)}\right)\right) \end{array}$$

**Figure 9:**
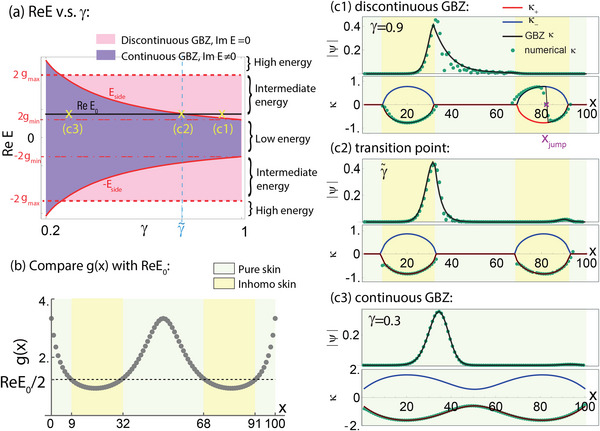
γ‐driven transition between continuous and discontinuous phase‐space GBZs. a) The phase diagram of a one‐component lattice with *g*(*x*) = 1/(sin (2π*x*/*L*)cos (π*x*/*L*) + 0.3) and *L* = 100, in the Re*E*‐γ parameter space. The curves ±*E*_side_, as calculated from Equation (21), separate the continuous and discontinuous phase‐space GBZ phases. For a fixed chosen value of Re*E*_0_ (black), one can always find the threshold value $\gamma =\tilde{\gamma }$ [Equation (47)] where the transition occurs. Here, *E*_0_ = *E*_side_ = 2.46 when γ is tuned to the value $\tilde{\gamma }=0.76$. b) For a eigenstate corresponding to *E*_0_, its pure/inhomogeneous skin regions correspond to positions where 2*g*(*x*) is above/below the value of Re *E*_0_. c) Illustrative states at various γ, correspond to (c1) the discontinuous GBZ phase, (c2) at the transition point and (c3) in the continuous GBZ phase. Clear‐cut regions of pure and inhomogeneous skins exist in the discontinuous GBZ and the transition point states. Within the inhomogeneous skin region, the local continuity of the GBZ ensures numerical |κ(*x*)| consistently adheres to one of |κ_±_(*x*)|, even near the sign‐inversion site. This enables the calculation of the single *x*_jump_ according to Equation (25).

It gives the value of γ across which a phase transition between continuous and discontinuous phase‐space GBZ occurs, as demonstrated in **Figure** **9**. The state *E*_0_ is real and resides within the discontinuous GBZ phase if $|\log\left(\gamma \right)|\leq |\log\left(\tilde{\gamma }\right)|$, but has to be complex otherwise.

Figure 9a shows the phase diagram of state *E*_0_ in the Re*E*‐γ space, where $\tilde{\gamma }\left(E_{0}\right)$ is determined as the point where Re*E*_0_ becomes the *E*_side_. For a fixed value of Re*E*_0_, $\tilde{\gamma }\left(E_{0}\right)$ separates the (c3) continuous (complex *E*_0_) and (c1,c2) discontinuous (real *E*_0_) phases. Crucially, the real state *E*_0_ within the discontinuous GBZ phase, and consequently its phase‐space GBZ solutions $\kappa_{n,\pm }$, remain robust against slight variation in γ, as verified by comparing (c1) and (c2). Adjusting γ solely shifts the discontinuity position *x*_jump_ along the real‐space position.

A similar threshold non‐Hermiticity $\tilde{\gamma }$ is defined for two‐component states with energy *E*_0_ within the lowest energy regime or the intermediate energy regime

(48) $$\begin{array}{cc} & \tilde{\gamma }\left(E_{0}\right)=\exp\left(-\frac{1}{L}\sum_{1<|\omega_{j}\left(\text{Re}\left(E_{0}\right),x\right)|}\cosh^{-1}\left|\omega_{j}\left(\text{Re}\left(E_{0}\right),x\right)\right|\right) \end{array}$$

where ${\tilde{\gamma }}_{\text{topo}}=\tilde{\gamma }\left(0\right)$ sets the threshold non‐Hermiticity for the topological zero mode.

### Handling Isolated Hopping Discontinuities

Here, the phase‐space GBZ formalism was extended to handle systems with local isolated non‐smoothness or discontinuity in the hoppings, such that they were treated as additional boundaries. Consider a non‐Hermitian inhomogeneous lattice with a discontinuous hopping function

(49) $$\begin{array}{cc} & g\left(x\right)=\left\{\begin{array}{cc} f_{1}\left(x\right) & \;\text{for}\;x_{0}\leq x<x_{1}\\ f_{2}\left(x\right) & \;\text{for}\;x_{1}\leq x<L+x_{0} \end{array}\right. \end{array}$$

where *f*_1, 2_(*x*) are smooth functions with *f*_1_(*x*_1_ − 1) ≠ *f*_2_(*x*_1_). Its eigenstate solution ψ(*x*) is solved by separately considering ψ^(1, 2)^(*x*)

(50) $$\begin{array}{cc} & \psi \left(x\right)=\left\{\begin{array}{cc} \psi^{\left(1\right)}\left(x\right) & \;\text{for}\;x_{0}\leq x\leq x_{1}\\ \psi^{\left(2\right)}\left(x\right) & \;\text{for}\;x_{1}\leq x\leq L+x_{0} \end{array}\right. \end{array}$$

with two boundary conditions at each discontinuous point

(51) $$\begin{array}{cc} & \psi^{\left(1\right)}\left(x_{0}\right)=\psi^{\left(2\right)}\left(x_{0}\right) \end{array}$$

(52) $$\begin{array}{cc} & f_{1}\left(x_{0}\right)\psi^{\left(1\right)}\left(x_{0}+1\right)=f_{2}\left(x_{0}\right)\psi^{\left(2\right)}\left(x_{0}+1\right) \end{array}$$

ψ^(1, 2)^(*x*) could be written as a superposition of phase‐space GBZ state solutions of both smooth‐hopping sub‐lattices simultaneously

(53) $$\begin{array}{cc} & \psi^{\left(1,2\right)}\left(x\right)=c_{+}^{\left(1,2\right)}\phi_{+}^{\left(1,2\right)}\left(x\right)+c_{-}^{\left(1,2\right)}\phi_{-}^{\left(1,2\right)}\left(x\right) \end{array}$$

with

(54) $$\begin{array}{cc} & \phi_{\pm }^{\left(1,2\right)}\left(x\right)=\frac{1}{\sqrt{f_{1,2}\left(x\right)}}\gamma^{x}\prod_{x^{\prime }=1}^{x}\beta_{n,\pm }^{\left(1,2\right)}\left(x\right) \end{array}$$

The superposed state solutions fulfill the bulk relation

(55) $$\begin{array}{cc} f_{1,2}\left(x\right) & /\gamma \psi^{\left(1,2\right)}\left(x+1\right)+f_{1,2}\left(x-1\right)\gamma \psi^{\left(1,2\right)}\left(x-1\right)=E_{n} \end{array}$$

for all *x* defined on *f*_1, 2_(*x*). In contrast to smooth‐hopping states that had only one of β_*n*, ±_(*x*) contributing to the wavefunction, each non‐smooth jump in *g*(*x*) required simultaneous contributions from both solutions, where one governs the overall trend and the other accounts for the fluctuation near the jump. Generally, two sub‐lattices with two non‐smooth jumps involve four non‐trivial coefficients $c_{\pm }^{\left(1,2\right)}$ to be solved

(56) $$\begin{array}{c} \text{Det}\left[\begin{array}{cccc} c_{+}^{\left(1\right)} & c_{-}^{\left(1\right)} & -c_{+}^{\left(2\right)} & -c_{-}^{\left(2\right)}\\ F_{1,+,+} & F_{1,-,-} & -F_{2,+,+} & -F_{2,-,-}\\ U_{1,+,+} & U_{1,-,-} & -U_{2,+,+} & -U_{2,-,-}\\ W_{1,+,+} & W_{1,-,-} & -W_{2,+,+} & -W_{2,-,-} \end{array}\right]=0 \end{array}$$

where

(57) $$\begin{array}{cc} F_{a,\mu ,\nu } & =f_{a}\left(x_{0}\right)c_{\mu }^{\left(a\right)}\gamma \beta_{n,\nu }^{\left(a\right)}\left(x_{0}+1\right) \end{array}$$

(58) $$\begin{array}{cc} U_{a,\mu ,\nu } & =c_{\mu }^{\left(a\right)}\prod_{x=x_{0}+1}^{x_{1}}\gamma \beta_{n,\nu }^{\left(a\right)}\left(x\right) \end{array}$$

(59) $$\begin{array}{cc} W_{a,\mu ,\nu } & =f_{a}\left(x_{1}\right)c_{\mu }^{\left(a\right)}\prod_{x=x_{0}+1}^{x_{1}+1}\gamma \beta_{n,\nu }^{\left(a\right)}\left(x\right) \end{array}$$

**Figure 10:**
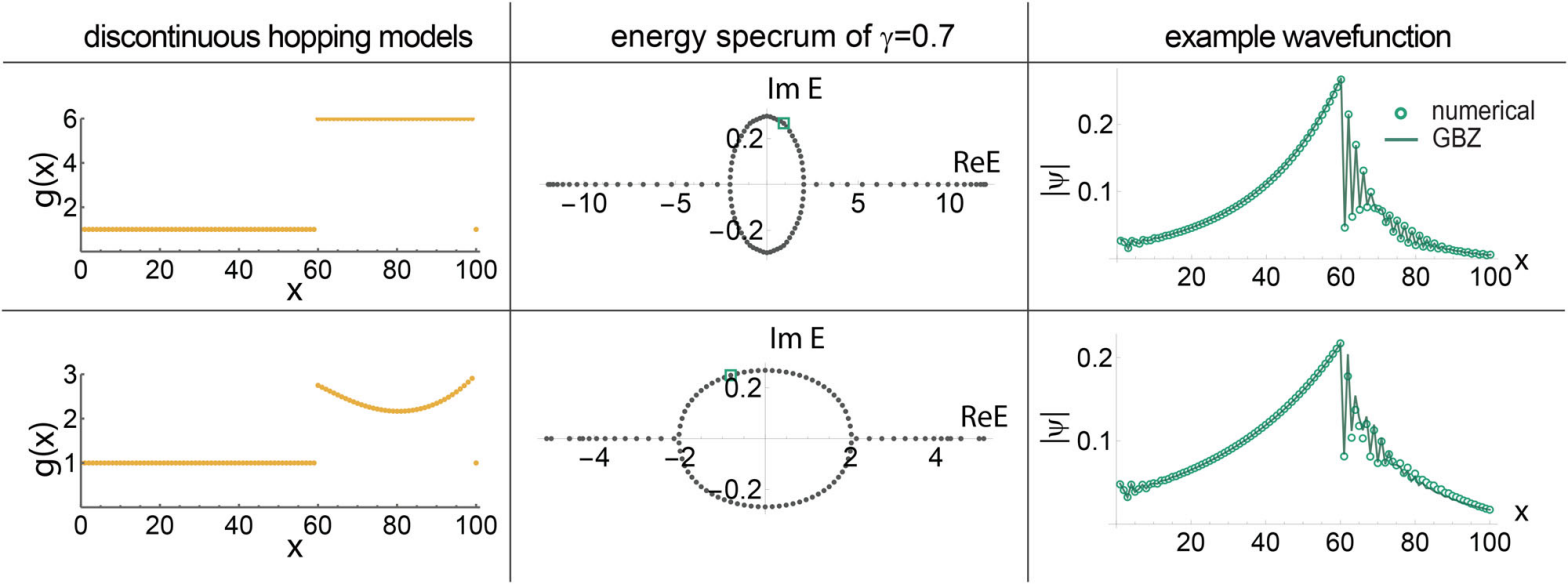
Incorporating isolated discontinuities in the hopping profiles into the phase‐space GBZ. Although the accuracy of the phase‐space GBZ approach may be reduced when non‐smoothness of *g*(*x*) is introduced, isolated non‐smoothness or discontinuities can be effectively addressed by matching superposed solutions at the discontinuity sites, as given by Equation (61). a) Two illustrative hopping profiles with discontinuities at *x* = 0 and $\frac{3L}{5}$, both with *g*(*x*) = 1 for $0\leq x<\frac{3L}{5}$. For the remaining lattice, the upper example has *g*(*x*) = 6 and the lower has *g*(*x*) = 1/(sin (2π*x*/*L*)cos (π*x*/*L*) + 2). b) Their spectra at γ = 0.7, both which exhibit characteristic real tails. c) By applying Equation (61) at *x* = 0 and $\frac{3L}{5}$ for two illustrative states (indicated by green hollow squares in (b)), the resultant wavefunction prediction (solid line) is seen to agree well with the numerical wavefunctions (circles) for both *g*(*x*) profiles.

In practice, with large *L*, strong NHSE accumulation occurs in one of the sub‐lattices due to the existence of inhomogeneous skin regions:

(60) $$\begin{array}{cc} & \prod_{x=x_{0}+1}^{x_{1}}\gamma \beta_{n,-}^{\left(j\right)}\left(x\right)\ll \prod_{x=x_{0}+1}^{x_{1}}\gamma \beta_{n,+}^{\left(j\right)}\left(x\right) \end{array}$$

and Equation (56) can be simplified by the approximation $c_{-}^{\left(j\right)}\to 0$, which is justified by Equation (60). For *j* = 1, Equation (56) reduces to

(61) $$\begin{array}{c} \text{Det}\left[\begin{array}{cc} U_{2,+,+}+U_{1,+,+} & U_{2,-,-}\\ W_{2,+,+}+W_{1,+,+} & W_{2,-,-} \end{array}\right]=0 \end{array}$$

whose solution gives all $c_{-}^{\left(j\right)}=0$ and completes the phase‐space GBZ construction for systems with isolated non‐smoothness. **Figure** **10** demonstrates examples of *g*(*x*) with a pair of discontinuities and how their states are well approximated by the GBZ approach with an additional discontinuity [Equation (61)].

### Detailed Derivation for the Two‐Component Phase‐Space Generalized Brillouin Zone (GBZ）

For completeness, the full derivation of the phase‐space GBZ formulation for spatially inhomogeneous two‐component chains was provided here.. This expands upon the concise presentation in Section 2.4, and closely follows the methodology used for the single‐component case in Section 2. We consider the Hamiltonian defined in Equation (27), acting on states of the form

(62) $$\Psi_{n}=\sum_{x}\left[\psi_{n,a}\left(x\right)\left|x,a\right\rangle +\psi_{n,b}\left(x\right)\left|x,b\right\rangle \right]$$

The time‐independent Schrödinger equation *H*Ψ_*n*_ = *E*_*n*_Ψ_*n*_ yields the following coupled recurrence relations:

(63) $$\begin{array}{cc} g_{1}\left(x\right)\frac{1}{\gamma_{1}}\psi_{n,b}\left(x\right)+g_{2}\left(x-1\right)\gamma_{2}\psi_{n,b}\left(x-1\right) & =E_{n}\psi_{n,a}\left(x\right) \end{array}$$

(64) $$\begin{array}{cc} g_{1}\left(x\right)\gamma_{1}\psi_{n,a}\left(x\right)+g_{2}\left(x\right)\frac{1}{\gamma_{2}}\psi_{n,a}\left(x+1\right) & =E_{n}\psi_{n,b}\left(x\right) \end{array}$$

Solutions satisfying periodic boundary conditions were sought:

(65) $$\psi_{n,j}\left(x+L\right)=\psi_{n,j}\left(x\right),\;\text{for}\;j=a,b$$

Inspired by the single‐component ansatz [Equation (3)], the two‐component ansatz is proposed:

(66) $$\psi_{n,j}\left(x\right)=\frac{{\left(\gamma_{1}\gamma_{2}\right)}^{x}}{\sqrt{g_{1}\left(x\right)g_{2}\left(x\right)}}\prod_{x^{\prime }=1}^{x}\beta_{n,j}\left(x^{\prime }\right)$$

with β_*n*, *j*_(*x*) encoding the non‐Bloch deformation at site *x* for sublattice *j* = *a*, *b*. Substituting Equation (66) into Equations (63)–(64) and assuming that *g*_1_(*x*), *g*_2_(*x*) are sufficiently smooth such that *g*_*j*_(*x* ± 1) ≈ *g*_*j*_(*x*) and $\beta_{n,j}^{\prime }\left(x\right)\ll \beta_{n,j}\left(x\right)$, a decoupled recurrence equation is obtained for each sublattice:

(67) $$\begin{array}{c} \frac{1}{2}\left(\beta_{n,j}\left(x\right)+\frac{1}{\beta_{n,j}\left(x\right)}\right)=\omega_{j}\left(E_{n},x\right) \end{array}$$

where

(68) $$\begin{array}{cc} \omega_{a}\left(E_{n},x\right) & =\frac{E_{n}^{2}-g_{1}^{2}\left(x\right)-g_{2}^{2}\left(x-1\right)}{2\sqrt{g_{1}\left(x-1\right)g_{1}\left(x\right)g_{2}\left(x-1\right)g_{2}\left(x\right)}} \end{array}$$

(69) $$\begin{array}{cc} \omega_{b}\left(E_{n},x\right) & =\frac{E_{n}^{2}-g_{1}^{2}\left(x\right)-g_{2}^{2}\left(x\right)}{2\sqrt{g_{1}\left(x\right)g_{1}\left(x+1\right)g_{2}\left(x-1\right)g_{2}\left(x\right)}} \end{array}$$

Solving Equation (67), the local GBZ branches for sublattice *j* take the form:

(70) $$\begin{array}{c} \beta_{n,j,\pm }\left(x\right)=\exp\left(\pm \cosh^{-1}\omega_{j}\left(E_{n},x\right)\right) \end{array}$$

(71) $$\begin{array}{c} \kappa_{n,j,\pm }\left(x\right)=\mp \text{Re}\left[\cosh^{-1}\omega_{j}\left(E_{n},x\right)\right] \end{array}$$

with κ_*n*, *j*, ±_(*x*) being the position‐dependent inverse skin depth. To determine the global constraint on *E*_*n*_, the PBC condition was enforced on the total wavefunction. Substituting Equation (66) into the periodicity requirement (Equation (65)) gives:

(72) $${\left(\gamma_{1}\gamma_{2}\right)}^{L}=\prod_{x=1}^{L}\beta_{n,j}\left(x\right),\;\text{for}\;j=a,b$$

which yields the spectral constraint:

(73) $$\log\left(\gamma_{1}\gamma_{2}\right)=-\frac{1}{L}\sum_{x=1}^{L}\sigma_{j}\left(x\right)\;\text{Re}\left[\cosh^{-1}\omega_{j}\left(E_{n},x\right)\right]$$

where σ_*j*_(*x*) = ±1 selects between the ± GBZ branches at each position. Finally, the spatial profile of the eigenstate amplitude for each component is:

(74) $$\begin{array}{c} |\psi_{n,j}\left(x\right)|=\frac{{\left(\gamma_{1}\gamma_{2}\right)}^{x}}{\sqrt{g_{1}\left(x\right)g_{2}\left(x\right)}}\prod_{x^{\prime }=1}^{x}\exp\left[-\kappa_{n,j}\left(x^{\prime }\right)\right] \end{array}$$

This completes the full derivation. As discussed in the main text, these expressions reduce to those of the single‐component case upon setting *g*_1_(*x*) = *g*_2_(*x*) = *g*(*x*) and γ_1_ = γ_2_.

## Conflict of Interest

The authors declare no conflict of interest.

## Data Availability

Data sharing is not applicable to this article as no new data were created or analyzed in this study.

## References

[1] S. Yao , Z. Wang , Phys. Rev. Lett. 2018, 121, 086803. 30192628 10.1103/PhysRevLett.121.086803

[2] K. Yokomizo , S. Murakami , Phys. Rev. Lett. 2019, 123, 066404. 31491170 10.1103/PhysRevLett.123.066404

[3] S. Longhi , Phys. Rev. Res. 2019, 1, 023013.

[4] F. Song , S. Yao , Z. Wang , Phys. Rev. Lett. 2019, 123, 246801. 31922829 10.1103/PhysRevLett.123.246801

[5] C. H. Lee , S. Longhi , Commun. Phys. 2020, 3, 147.

[6] K. Zhang , Z. Yang , C. Fang , Phys. Rev. Lett. 2020, 125, 126402. 33016766 10.1103/PhysRevLett.125.126402

[7] K. Kawabata , N. Okuma , M. Sato , Phys. Rev. B 2020, 101, 195147. 10.1103/PhysRevLett.124.086801 32167324

[8] H. Jiang , C. H. Lee , Phys. Rev. Lett. 2023, 131, 076401. 37656848 10.1103/PhysRevLett.131.076401

[9] H.‐Y. Wang , F. Song , Z. Wang , Phys. Rev. X 2024, 14, 021011.

[10] K. Yokomizo , T. Yoda , Y. Ashida , Phys. Rev. B 2024, 109, 115115.

[11] C.‐H. Liu , K. Zhang , Z. Yang , S. Chen , Phys. Rev. Res. 2020, 2, 043167.

[12] C. H. Lee , Phys. Rev. Lett. 2022, 128, 010402. 35061451 10.1103/PhysRevLett.128.010402

[13] F. Qin , Y. Ma , R. Shen , C. H. Lee , Phys. Rev. B 2023, 107, 155430.

[14] S. Rafi‐Ul‐Islam , Z. B. Siu , H. Sahin , C. H. Lee , M. B. Jalil , Phys. Rev. Res. 2022, 4, 013243.

[15] M. Yang , C. H. Lee , Phys. Rev. Lett. 2024, 133, 136602. 39392962 10.1103/PhysRevLett.133.136602

[16] K. Yokomizo , S. Murakami , Phys. Rev. B 2021, 104, 165117.

[17] R. Lin , T. Tai , L. Li , C. H. Lee , Front. Phys. 2023, 18, 53605.

[18] S. Liu , H. Jiang , W.‐T. Xue , Q. Li , J. Gong , X. Liu , C. H. Lee , Sci. Bull. 2025, 70. 10.1016/j.scib.2025.07.011 40796459

[19] L. Li , C. H. Lee , Sci. Bull. 2022, 67, 685. 10.1016/j.scib.2022.01.017 36546132

[20] G.‐F. Guo , X.‐X. Bao , L. Tan , New J. Phys. 2021, 23, 123007.

[21] Z. Yang , K. Zhang , C. Fang , J. Hu , Phys. Rev. Lett. 2020, 125, 226402. 33315431 10.1103/PhysRevLett.125.226402

[22] D. S. Borgnia , A. J. Kruchkov , R.‐J. Slager , Phys. Rev. Lett. 2020, 124, 056802. 32083895 10.1103/PhysRevLett.124.056802

[23] R. Ye , Y. He , G. Li , L. Wang , X. Wu , X. Qiao , Y. Zheng , L. Jin , D.‐W. Wang , L. Yuan , X. Chen , Light: Sci. Appl. 2025, 14, 39. 39774943 10.1038/s41377-024-01700-1 PMC11707151

[24] N. Okuma , M. Sato , Annu. Rev. Condens. Matter Phys. 2023, 14, 83.

[25] D. Nakamura , T. Bessho , M. Sato , Phys. Rev. Lett. 2024, 132, 136401. 38613277 10.1103/PhysRevLett.132.136401

[26] K. Zhang , Z. Yang , C. Fang , Nat. Commun. 2022, 13, 2496. 35523795 10.1038/s41467-022-30161-6 PMC9076925

[27] C. H. Lee , L. Li , J. Gong , Phys. Rev. Lett. 2019, 123, 016805. 31386404 10.1103/PhysRevLett.123.016805

[28] H. Zhang , T. Chen , L. Li , C. H. Lee , X. Zhang , Phys. Rev. B 2023, 107, 085426.

[29] N. Okuma , K. Kawabata , K. Shiozaki , M. Sato , Phys. Rev. Lett. 2020, 124, 086801. 32167324 10.1103/PhysRevLett.124.086801

[30] L. Li , C. H. Lee , J. Gong , Phys. Rev. Lett. 2020, 124, 250402. 32639752 10.1103/PhysRevLett.124.250402

[31] L. Li , S. Mu , C. H. Lee , J. Gong , Nat. Commun. 2021, 12, 5294. 34489421 10.1038/s41467-021-25626-z PMC8421445

[32] X.‐Q. Sun , P. Zhu , T. L. Hughes , Phys. Rev. Lett. 2021, 127, 066401. 34420349 10.1103/PhysRevLett.127.066401

[33] R. Shen , C. H. Lee , Commun. Phys. 2022, 5, 238.

[34] M.‐H. L. Xiujuan Zhang , Tian Zhang , Y.‐F. Chen , Adv. Phys.: X 2022, 7, 2109431.

[35] G.‐F. Guo , X.‐X. Bao , H.‐J. Zhu , X.‐M. Zhao , L. Zhuang , L. Tan , W.‐M. Liu , Commun. Phys. 2023, 6, 363.

[36] T. Yoshida , S.‐B. Zhang , T. Neupert , N. Kawakami , Phys. Rev. Lett. 2024, 133, 076502. 39213584 10.1103/PhysRevLett.133.076502

[37] C. Shang , S. Liu , R. Shao , P. Han , X. Zang , X. Zhang , K. N. Salama , W. Gao , C. H. Lee , R. Thomale , A. Manchon , S. Zhang , T. J. Cui , U. Schwingenschlögl , Adv. Sci. 2022, 9, 2202922. 10.1002/advs.202202922 PMC9799024 36372546

[38] Z. Lei , C. H. Lee , L. Li , Commun. Phys. 2024, 7, 100.

[39] R. Shen , T. Chen , B. Yang , C. H. Lee , Nat. Commun. 2025, 16, 1340. 39905021 10.1038/s41467-025-55953-4 PMC11794467

[40] Y. Qin , C. H. Lee , L. Li , Commun. Phys. 2025, 8, 18.

[41] L. Li , C. H. Lee , J. Gong , Commun. Phys. 2021, 4, 42.

[42] C.‐X. Guo , X. Wang , H. Hu , S. Chen , Phys. Rev. B 2023, 107, 134121.

[43] B. Li , H.‐R. Wang , F. Song , Z. Wang , Phys. Rev. B 2023, 108, L161409.

[44] H.‐R. Wang , B. Li , F. Song , Z. Wang , SciPost Phys. 2023, 15, 191.

[45] X. Xie , G. Liang , F. Ma , Y. Du , Y. Peng , E. Li , H. Chen , L. Li , F. Gao , H. Xue , Phys. Rev. B 2024, 109, L140102.

[46] G.‐J. Liu , J.‐M. Zhang , S.‐Z. Li , Z. Li , Phys. Rev. A 2024, 110, 012222.

[47] R. Wang , K. Zhang , Z. Song , J. Phys. Commun. 2021, 5, 095011.

[48] S. Longhi , Phys. Rev. Lett. 2019, 122, 237601. 31298877 10.1103/PhysRevLett.122.237601

[49] S. Longhi , Opt. Lett. 2023, 48, 6251. 38039239 10.1364/OL.507937

[50] Q.‐B. Zeng , R. Lü , New J. Phys. 2022, 24, 043023.

[51] H. Wang , W. Zhang , H. Sun , X. Zhang , Phys. Rev. B 2023, 108, 144203.

[52] S. Longhi , Opt. Lett. 2024, 49, 1373. 38427016 10.1364/OL.517182

[53] Z. Chen , K. Kawabata , A. Kulkarni , S. Ryu , Phys. Rev. B 2025, 111, 054203.

[54] S. Longhi , Opt. Lett. 2025, 50, 746. 39888742 10.1364/OL.551954

[55] T. Liu , H. Guo , Y. Pu , S. Longhi , Phys. Rev. B 2020, 102, 024205.

[56] S. Longhi , Phys. Rev. Lett. 2020, 124, 066602. 32109127 10.1103/PhysRevLett.124.066602

[57] S. Longhi , Opt. Lett. 2022, 47, 6345. 36538434 10.1364/OL.478059

[58] R. Qi , J. Cao , X.‐P. Jiang , arXiv preprint arXiv:2306.03807 2023.

[59] F. K. Kunst , E. Edvardsson , J. C. Budich , E. J. Bergholtz , Phys. Rev. Lett. 2018, 121, 026808. 30085697 10.1103/PhysRevLett.121.026808

[60] S. Yao , F. Song , Z. Wang , Phys. Rev. Lett. 2018, 121, 136802. 30312068 10.1103/PhysRevLett.121.136802

[61] C. H. Lee , R. Thomale , Phys. Rev. B 2019, 99, 201103.

[62] F. Song , S. Yao , Z. Wang , Phys. Rev. Lett. 2019, 123, 170401. 31702238 10.1103/PhysRevLett.123.170401

[63] C. H. Lee , L. Li , R. Thomale , J. Gong , Phys. Rev. B 2020, 102, 085151.

[64] Z. Gong , Y. Ashida , K. Kawabata , K. Takasan , S. Higashikawa , M. Ueda , Phys. Rev. X 2018, 8, 031079.

[65] R. Yang , J. W. Tan , T. Tai , J. M. Koh , L. Li , S. Longhi , C. H. Lee , Sci. Bull. 2022, 67, 1865. 10.1016/j.scib.2022.08.005 36546300

[66] T. Tai , C. H. Lee , Phys. Rev. B 2023, 107, L220301.

[67] X. Zhang , B. Zhang , W. Zhao , C. H. Lee , SciPost Phys. 2024, 16, 002.

[68] L. Li , C. H. Lee , S. Mu , J. Gong , Nat. Commun. 2020, 11, 5491. 33127908 10.1038/s41467-020-18917-4 PMC7603343

[69] In more complicated spatially‐inhomogeneous lattices with further hoppings, which we will not consider in this work, not only will there be more β_ *n* _(*x*) branches, γ would also depend on the momentum.^[8,12,13,22,28–34,63]^

[70] For instance, the spectrum of a spatially homogeneous Hatano Nelson model is an ellipse in the complex energy plane, and only two isolated ±*E* _side_ = ±*g*(γ + γ^−1^) energies are real.

[71] S. Manna , B. Roy , Commun. Phys. 2023, 6, 10.

[72] H. Shen , B. Zhen , L. Fu , Phys. Rev. Lett. 2018, 120, 146402. 29694133 10.1103/PhysRevLett.120.146402

[73] X. Chen , Z.‐C. Gu , Z.‐X. Liu , X.‐G. Wen , Phys. Rev. B 2013, 87, 155114.

[74] C.‐K. Chiu , J. C. Teo , A. P. Schnyder , S. Ryu , Rev. Mod. Phys. 2016, 88, 035005.

[75] X.‐G. Wen , in Topological Phase Transitions and New Developments, Springer, Berlin 2017, pp. 163–189.

[76] X. Chen , Z.‐C. Gu , X.‐G. Wen , Phys. Rev. B 2011, 83, 035107.

[77] C. H. Lee , S. Imhof , C. Berger , F. Bayer , J. Brehm , L. W. Molenkamp , T. Kiessling , R. Thomale , Commun. Phys. 2018, 1, 39.

[78] T. Helbig , T. Hofmann , C. H. Lee , R. Thomale , S. Imhof , L. W. Molenkamp , T. Kiessling , Phys. Rev. B 2019, 99, 161114.

[79] Y. Wang , B. Zhang , Y. Chong , Nat. Commun. 2020, 11, 2846. 32398727 10.1038/s41467-020-15940-3 PMC7217906

[80] T. Hofmann , T. Helbig , F. Schindler , N. Salgo , M. Brzezińska , M. Greiter , T. Kiessling , D. Wolf , A. Vollhardt , A. Kabaši , R. Thomale , T. Neupert , Phys. Rev. Res. 2020, 2, 023265.

[81] D. Zou , T. Chen , W. He , J. Bao , C. H. Lee , H. Sun , X. Zhang , Nat. Commun. 2021, 12, 7201. 34893589 10.1038/s41467-021-26414-5 PMC8664810

[82] L. Su , H. Jiang , Z. Wang , S. Chen , D. Zheng , Phys. Rev. B 2023, 107, 184108.

[83] H. Hohmann , T. Hofmann , T. Helbig , S. Imhof , H. Brand , L. K. Upreti , A. Stegmaier , A. Fritzsche , T. Müller , U. Schwingenschlögl , C. H. Lee , M. Greiter , L. W. Molenkamp , T. Kießling , R. Thomale , Phys. Rev. Res. 2023, 5, L012041.

[84] D. Zou , T. Chen , H. Meng , Y. S. Ang , X. Zhang , C. H. Lee , Sci. Bull. 2024, 69, 2194. 10.1016/j.scib.2024.05.036 38853044

[85] C.‐X. Guo , L. Su , Y. Wang , L. Li , J. Wang , X. Ruan , Y. Du , D. Zheng , S. Chen , H. Hu , Nat. Commun. 2024, 15, 9120. 39438469 10.1038/s41467-024-53434-8 PMC11496883

[86] J. Li , A. K. Harter , J. Liu , L. de Melo , Y. N. Joglekar , L. Luo , Nat. Commun. 2019, 10, 855. 30787299 10.1038/s41467-019-08596-1 PMC6382795

[87] Z. Ren , D. Liu , E. Zhao , C. He , K. K. Pak , J. Li , G.‐B. Jo , Nat. Phys. 2022, 18, 385.

[88] Q. Liang , D. Xie , Z. Dong , H. Li , H. Li , B. Gadway , W. Yi , B. Yan , Phys. Rev. Lett. 2022, 129, 070401. 36018690 10.1103/PhysRevLett.129.070401

[89] L. Zhou , H. Li , W. Yi , X. Cui , Commun. Phys. 2022, 5, 252.

[90] W. Song , S. Wu , C. Chen , Y. Chen , C. Huang , L. Yuan , S. Zhu , T. Li , Phys. Rev. Lett. 2023, 130, 043803. 36763423 10.1103/PhysRevLett.130.043803

[91] Z. Lin , L. Ding , S.‐K. Ke , X. Li , Opt. Lett. 2021, 46, 3512. 34329212 10.1364/OL.431904

[92] F. Roccati , M. Bello , Z. Gong , M. Ueda , F. Ciccarello , A. Chenu , A. Carollo , Nat. Commun. 2024, 15, 2400. 38493191 10.1038/s41467-024-46471-w PMC10944496

[93] H. Kamakari , S.‐N. Sun , M. Motta , A. J. Minnich , PRX Quantum 2022, 3, 010320.

[94] J. M. Koh , S.‐N. Sun , M. Motta , A. J. Minnich , Nat. Phys. 2023, 19, 1314.

[95] E. Chertkov , Z. Cheng , A. C. Potter , S. Gopalakrishnan , T. M. Gatterman , J. A. Gerber , K. Gilmore , D. Gresh , A. Hall , A. Hankin , M. Matheny , T. Mengle , D. Hayes , B. Neyenhuis , R. Stutz , M. Foss‐Feig , Nat. Phys. 2023, 19, 1799.

[96] J. M. Koh , T. Tai , C. H. Lee , Nat. Commun. 2024, 15, 5807. 38987264 10.1038/s41467-024-49648-5 PMC11237062

[97] J. M. Koh , T. Tai , C. H. Lee , Phys. Rev. Lett. 2022, 129, 140502. 36240412 10.1103/PhysRevLett.129.140502

[98] R. Shen , F. Qin , J.‐Y. Desaules , Z. Papić , C. H. Lee , Phys. Rev. Lett. 2024, 133, 216601. 39642519 10.1103/PhysRevLett.133.216601

[99] L. Xiao , W.‐T. Xue , F. Song , Y.‐M. Hu , W. Yi , Z. Wang , P. Xue , Phys. Rev. Lett. 2024, 133, 070801. 39213575 10.1103/PhysRevLett.133.070801

[100] Z. Lan , W. Liu , Y. Wu , X. Ye , Z. Yang , C.‐K. Duan , Y. Wang , X. Rong , Chin. Phys. Lett. 2024, 41, 050301.

[101] M. Yang , L. Wang , X. Wu , H. Xiao , D. Yu , L. Yuan , X. Chen , Phys. Rev. A 2022, 106, 043717.

[102] D. Braghini , L. G. G. Villani , M. I. N. Rosa , J. R. de F Arruda , J. Phys. D: Appl. Phys. 2021, 54, 285302.

[103] Y. Jin , W. Zhong , R. Cai , X. Zhuang , Y. Pennec , B. Djafari‐Rouhani , Appl. Phys. Lett. 2022, 121, 022202.

[104] M. Ezawa , Phys. Rev. B 2019, 100, 075423.

[105] M. Ezawa , Phys. Rev. B 2019, 100, 081401.

[106] Y. Lu , N. Jia , L. Su , C. Owens , G. Juzeliūnas, D. I. Schuster , J. Simon , Commun. Phys. 2023, 6, 1404.

[107] X. Zhang , K. Ding , X. Zhou , J. Xu , D. Jin , Phys. Rev. B 2022, 105, 195127.

[108] S. Franca , T. Seidemann , F. Hassler , J. van den Brink , I. C. Fulga , Phys. Rev. B 2024, 109, L241103.

